# Detailed phenotyping identifies genes with pleiotropic effects on body composition

**DOI:** 10.1186/s12864-016-2538-0

**Published:** 2016-03-12

**Authors:** Sunduimijid Bolormaa, Ben J. Hayes, Julius H.J. van der Werf, David Pethick, Michael E. Goddard, Hans D. Daetwyler

**Affiliations:** AgriBio, Centre for AgriBioscience, Department of Economic Development, Jobs, Transport and Resources, Bundoora, VIC 3083 Australia; Cooperative Research Centre for Sheep Industry Innovation, Armidale, NSW 2351 Australia; School of Applied Systems Biology, La Trobe University, Bundoora, VIC 3083 Australia; School of Environmental and Rural Science, University of New England, Armidale, NSW 2351 Australia; School of Veterinary and Life Sciences, Murdoch University, Murdoch, WA 6150 Australia; School of Land and Environment, University of Melbourne, Parkville, VIC 3010 Australia

**Keywords:** GWAS, Multi-trait, Meta-analysis, Pleiotropy, Genes, Body composition, Sheep, Human

## Abstract

**Background:**

Genetic variation in both the composition and distribution of fat and muscle in the body is important to human health as well as the healthiness and value of meat from cattle and sheep. Here we use detailed phenotyping and a multi-trait approach to identify genes explaining variation in body composition traits.

**Results:**

A multi-trait genome wide association analysis of 56 carcass composition traits measured on 10,613 sheep with imputed and real genotypes on 510,174 SNPs was performed. We clustered 71 significant SNPs into five groups based on their pleiotropic effects across the 56 traits. Among these 71 significant SNPs, one group of 11 SNPs affected the fatty acid profile of the muscle and were close to 8 genes involved in fatty acid or triglyceride synthesis. Another group of 23 SNPs had an effect on mature size, based on their pattern of effects across traits, but the genes near this group of SNPs did not share any obvious function. Many of the likely candidate genes near SNPs with significant pleiotropic effects on the 56 traits are involved in intra-cellular signalling pathways. Among the significant SNPs were some with a convincing candidate gene due to the function of the gene (e.g. glycogen synthase affecting glycogen concentration) or because the same gene was associated with similar traits in other species.

**Conclusions:**

Using a multi-trait analysis increased the power to detect associations between SNP and body composition traits compared with the single trait analyses. Detailed phenotypic information helped to identify a convincing candidate in some cases as did information from other species.

**Electronic supplementary material:**

The online version of this article (doi:10.1186/s12864-016-2538-0) contains supplementary material, which is available to authorized users.

## Background

Body composition, including the distribution and composition of fat and muscle, is of great interest in a number of species. In humans it is predictive of health outcomes, and in cattle and sheep it affects the efficiency of meat production and the value and healthiness of the meat. Genetic variation in these traits is well documented. In humans the heritability of body mass index has been estimated to be 0.42 [1]. The genetic architecture of body mass index (BMI) appears to be highly polygenic, with the 97 loci of largest estimated effect accounting for ∼ 2.7 % of BMI variance [2]. In cattle and sheep, carcass composition traits have similar heritabilities to BMI in humans [3–5].

Genome wide association studies have been very successful at finding genetic variants, such as single nucleotide polymorphisms (SNPs), associated with body size and composition in humans and livestock [1, 2, 6, 7]. In some cases, the gene through which the causal variant acts is clear and the physiological pathway to the phenotype is at least partly understood. However, sometimes the causal gene is ambiguous and often the physiological pathway is almost unknown. For instance, genetic variants near the gene *LCORL* have been implicated as affecting size in several species [6–9], but the mechanism by which they do this is unknown.

In identifying the genes involved and the mechanism by which they act, an advantage of studies in animal species is that more detailed phenotypes relating to body composition can be collected. For example, these can include the amounts of bone, muscle and fat and the chemical composition of the fat and muscle. Since common physiological mechanisms (e.g. growth hormone) affect the growth of muscle, fat and bone and presumably the detailed composition of fat and muscle, detailed phenotypes should help to clarify the mechanism by which genetic variants affect these traits. For instance, selection for leanness and muscling led to reduced muscle oxidative capacity and iron concentration implying a change in the proportions of different muscle fibre types [10].

In this study, we did not record muscle fibre type directly but did record phenotypes associated with fibre type. Muscles vary in muscle fibre type composition [11]. Muscles with a predominance of slow twitch (type 1) fibres have a higher capacity for fatty acid uptake and lipid oxidation, have abundant mitochondria, and are rich in myoglobin that is responsible for the red colour of meat. Whereas, muscles requiring rapid contraction generating substantial force, such as some locomotory muscles, have a greater proportion of fast twitch fibres (type IIb and IIx), which largely depend on glycolytic metabolism for energy generation and are whiter and have fewer mitochondria [12, 13]. These muscles have lower activity of isocitrate dehydrogenase (ICDH) which is crucial in the oxygen-dependent citric acid cycle of mitochondria [14]. The rate of protein turnover in muscle fibres, pre- and post-slaughter, affects the growth of muscle and the tenderness of the meat. For instance, in sheep the callipyge mutation increases muscle in the trunk and hind quarters and decreases tenderness [15, 16]. Thus, it is likely that genetic variants affect multiple traits associated with the growth of muscle and fat and their detailed composition.

It is often suggested that detailed phenotyping will aid the identification of genetic variants affecting complex traits and help to elucidate the pathway by which they act. Here we test this hypothesis by phenotyping sheep for 56 traits associated with muscle and fat growth and composition.

Usually GWAS, for instance on human height and BMI, are analysed one trait at a time ignoring patterns of pleiotropy amongst these traits. However, the pattern of pleiotropy may help to identify the gene underlying an association and the mechanism through which it acts. Bolormaa et al. [17] showed that a multi-trait analysis, by combining the results from GWAS on 32 individual traits in beef cattle, increased the power to detect pleiotropic QTL. They also showed that cluster analysis identified groups of QTL with similar patterns of pleiotropic effects. It would help us to identify the genes underlying QTL and to understand their mechanisms of action, if these groups of QTL represent genes with similar function or belonging to the same pathways.

In this study we have analysed 56 detailed body composition phenotypes on 10,613 sheep with genotypes for 510,174 SNPs. We have tested four hypotheses:

- that a multi-trait analysis increases power to detect and map QTL,
- that detailed phenotyping increases power to identify the causal gene underlying the QTL and the mechanism by which it acts,
- that variants in different genes that act in the same pathway have a similar pattern of effects across traits, and
- that variants in the same genes affect growth and body composition in multiple species.

## Results

### Single-trait genome-wide association studies

**Fig. 1:**
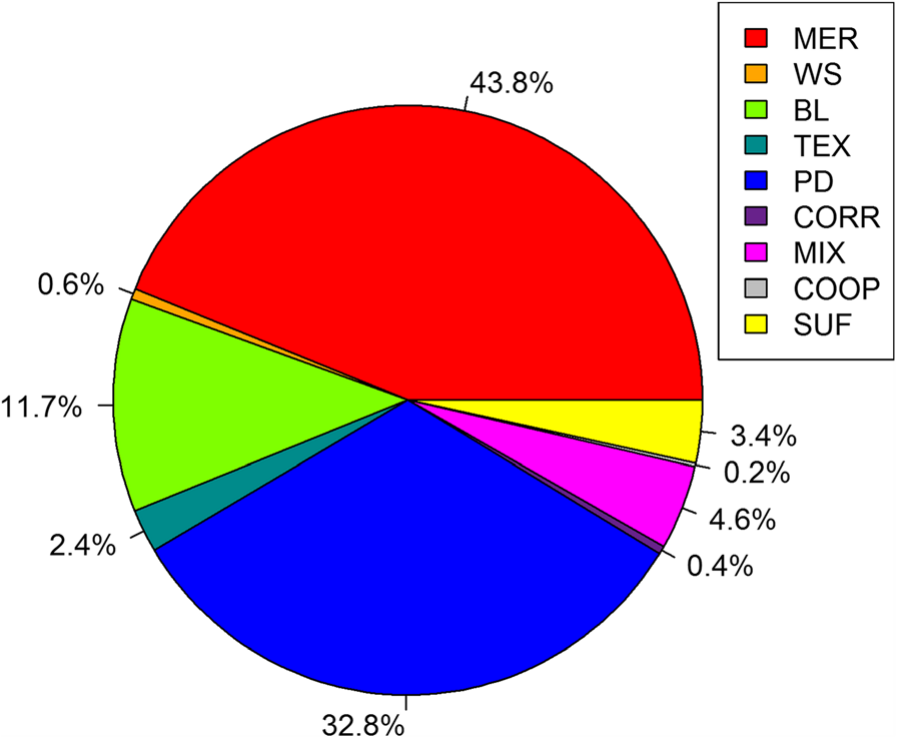
Pie chart showing percentages of total of 10,613 animals in each of sheep populations

In this study, 10,613 sheep that had real or imputed genotypes for 510,174 SNPs, were measured for up to 56 traits (Table 1), and belonged to a large number of breeds (Merino, **MER**; Poll Dorset, **PD**; Border Leicester, **BL**; Suffolk, **SUF**; white Suffolk, **WS**; Texel, **TEX**; Corriedale, **CORR**; Coopworth, **COOP**; and various Crosses, **MIX**; Fig. 1). GWAS, in which each SNP was fitted one at time, were performed for 56 traits including carcass weight, fatness, muscling, tenderness, meat colour, and fatty acid (FA) composition of fat (Table 1). Population structure was accounted for by fitting breed composition and a multi-generation pedigree. Table 2 presents the number of significant (*P* < 10^−5^ and *P* < 5 × 10^−7^) SNPs. Twenty nine traits had more than 10 significant SNPs (*P* < 5 × 10^−7^), resulting in a false discovery rate (**FDR**) of less than 2.6 % (Table 2).

**Table 1:** Number of records, mean, standard deviation (SD) and heritability estimate (h^2^) of each trait for the genotyped animals

Trait code (unit)	No.	Mean	SD	V_p_	h^2^	Trait name
PSWT (kg)	9193	48.5	7.3	17.8	0.23	Slaughter weight
HCWT (kg)	10428	22.1	4.1	5.3	0.27	Hot carcass weight
LLWT (g)	8226	357.9	68.9	2001.9	0.33	Loin weight
TOP (g)	8228	598.3	101.0	3137.5	0.31	Topside weight
RND (g)	8229	457.7	73.7	1833.8	0.32	Round weight
BONE (g)	8225	940.9	139.4	6508.5	0.31	leg + aitch bone weight
LEGBONE (g)	7472	547.7	68.9	2051.3	0.48	Leg bone weight
LMY (%)	9797	53.4	9.7	4.6	0.26	Lean meat yield
DRESSING (%)	9024	45.1	3.7	5.4	0.37	Dressing%
DMLOIN (%)	8035	26.7	1.1	0.8	0.26	Dry matter
IMF (%)	8242	4.3	1.0	0.7	0.51	Intramuscular fat
LLFAT (g)	8215	200.1	103.5	3071.1	0.17	Loin fat weight
CFATSCORE	5224	3.0	1.0	0.3	0.12	Carcass fat score
CCFAT (mm)	10183	4.0	2.3	2.8	0.21	Fat Depth C
HGRFAT (mm)	8384	13.2	5.5	10.0	0.34	Fat depth GR
CFAT5 (mm)	8132	7.0	3.4	5.1	0.18	Fat Depth 5th rib
CEMW (mm)	10343	60.2	5.2	14.4	0.35	Carcass eye muscle width
CEMD (mm)	10345	29.0	4.5	8.4	0.16	Carcass eye muscle depth
CEMA (cm^2^)	10345	14.0	2.9	3.1	0.24	Eye muscle area
SHEARF5 (N)	9991	29.1	10.6	63.0	0.25	Shear force day 5
SHEARF1 (N)	5325	41.3	14.3	103.5	0.28	Shear force day 1
MYOGLOBIN (mg/g wet)	8138	6.3	1.8	1.3	0.25	Myoglobin
GLYCOGEN (mmol/kg)	3116	61.3	14.1	140.7	0.16	Glycogen
ICDH activity (umol/min/g wet)	4742	4.6	1.7	0.9	0.27	ICDH activity
RCL4	4631	36.7	2.9	4.2	0.43	Retail colour^a^ L day 4
RCL3	4757	36.7	2.8	4.2	0.40	Retail colour^a^ L day 3
RCL2	4756	37.0	2.8	4.1	0.35	Retail colour^a^ L day 2
RCL1	4756	35.0	3.2	4.3	0.27	Retail colour^a^ L day 1
RCb4	4631	16.7	2.5	1.7	0.11	Retail colour^a^ b day 4
RCb3	4753	17.6	2.5	2.1	0.10	Retail colour^a^ b day 3
RCb2	4755	18.4	2.4	2.1	0.04	Retail colour^a^ b day 2
RCb1	4750	15.2	2.4	2.1	0.08	Retail colour^a^ b day 1
RCa4	4630	15.6	2.2	2.2	0.29	Retail colour^a^ a day 4
RCa3	4757	16.7	2.5	2.8	0.27	Retail colour^a^ a day 3
RCa2	4756	18.2	2.8	3.9	0.22	Retail colour^a^ a day 2
RCa1	4756	17.0	2.2	2.1	0.18	Retail colour^a^ a day 1
CFb	9764	4.6	4.1	1.3	0.13	Fresh colour b*
CFa	9758	16.0	5.3	1.7	0.08	Fresh colour a*
CFL	9754	34.4	3.1	4.2	0.21	Fresh colour L*
PH24LL	10299	5.7	0.2	0.0	0.16	LL pH 24 h
PH24ST	8223	5.8	0.2	0.0	0.19	ST pH 24 h
IRON (wet, mg/kg)	8083	20.0	3.4	7.3	0.24	Iron (wet)
ZINC (wet, mg/kg)	8099	24.9	4.6	16.7	0.22	Zinc (wet)
EPADPADHA (mg/100g^b^)	6539	48.4	16.9	69.2	0.14	EPA^c1^ + DPA^c2^ + DHA^c3^
EPADHA (mg/100g^b^)	6536	23.5	10.4	23.1	0.18	EPA^c1^ + DHA^c3^
FA_C22_6n3 (mg/100g^b^)	8140	6.9	3.2	3.5	0.26	DHA^c3^ (C22:6n3)
FA_C22_5n3 (mg/100g^b^)	8142	24.1	9.2	21.3	0.08	DPA^c2^ (C22:5n3)
FA_C20_5n3 (mg/100g^b^)	8141	15.4	7.9	13.4	0.13	EPA^c1^ (C20:5n3)
FA_C20_4n6 (mg/100g^b^)	8139	45.7	14.9	57.5	0.16	Arachidonic acid (C20:4n6)
FA_C20_3n6 (mg/100g^b^)	8136	4.5	1.4	0.8	0.16	DGLA^c4^ (C20:3n6)
FA_C18_2n6 (mg/100g^b^)	8130	134.7	40.2	482.1	0.15	Linoleic acid (C18:2n6)
FA_C18_0 (mg/100g^b^)	8124	481.3	161.5	9903.2	0.19	Stearic acid (C18:0)
FA_C16_0 (mg/100g^b^)	8131	650.9	224.0	17030.0	0.11	Palmitic acid (C16:0)
FA_C14_0 (mg/100g^b^)	8128	70.2	29.5	402.6	0.15	Myristic acid (C14:0)
FA_C12_0 (mg/100g^b^)	8116	4.3	2.4	2.7	0.13	Lauric acid (C12:0)
FA_C10_0 (mg/100g^b^)	8108	4.5	2.3	2.5	0.11	Capric acid (C10:0)

^a^retail colour trait using HunterLab colour meter, ^b^mg/100 g wet muscle tissue, ^c1^Eicosapentaenoic acid, ^c2^Docosapentaenoic acid, ^c3^Docosahexaenoic acid, ^c4^Dihomo-γ-linolenic acid

**Table 2:** Number of SNPs and their false discovery rates (%) for each trait before^b^ (*P* < 5 × 10^−7^ and *P* < 5 × 10^−7^) and after^c^ (*P* < 5 × 10^−7^) fitting the 23 lead SNPs in the model

	*P* < 1 × 10^−5^		*P* < 5 × 10^−7^			
Trait^a^	No.^b^	FDR^b^	No.^b^	FDR^b^	No.^c^	FDR^c^
PSWT	245	2.1	105	0.2	44	0.6
HCWT	156	3.3	24	1.1	18	1.4
LLWT	64	8.0	28	0.9	12	2.1
TOP	58	8.8	10	2.6		
RND	158	3.2	83	0.3	7	3.6
BONE	565	0.9	379	0.1	165	0.2
LEGBONE	888	0.6	626	0.04	596	0.04
LMY	256	2.0	125	0.2	12	2.1
DRESSING	256	2.0	112	0.2	14	1.8
DMLOIN	82	6.2	30	0.9	2	12.8
IMF	58	8.8	13	2.0	5	5.1
LLFAT	120	4.3	58	0.4	7	3.6
CFATSCORE	13	39.2				
CCFAT	223	2.3	122	0.2	12	2.1
HGRFAT	275	1.9	167	0.2	18	1.4
CFAT5	43	11.9	9	2.8	1	25.5
CEMW	88	5.8	20	1.3	3	8.5
CEMD	23	22.2	2	12.8		
CEMA	32	15.9	6	4.3	3	8.5
SHEARF5	37	13.8	13	2.0	3	8.5
SHEARF1	19	26.9	2	12.8		
MYOGLOBIN	37	13.8	13	2.0	2	12.8
GLYCOGEN	52	9.8	20	1.3	2	12.8
ICDHACTIVITY	75	6.8	35	0.7	6	4.3
RCL4	41	12.4	10	2.6	3	8.5
RCL3	21	24.3	6	4.3	1	25.5
RCL2	19	26.9	3	8.5	0	
RCL1	12	42.5	0		0	
RCb4	6	85.0	0		0	
RCb3	9	56.7	0		0	
RCb2	5	102.0	0		0	
RCb1	19	26.9	2	12.8	0	
RCa4	48	10.6	18	1.4	0	
RCa3	55	9.3	24	1.1	0	
RCa2	57	8.9	29	0.9	0	
RCa1	68	7.5	23	1.1	1	25.5
CFb	6	85.0	0		0	
CFa	36	14.2	2	12.8	3	8.5
CFL	18	28.3	4	6.4	23	1.1
PH24LL	154	3.3	89	0.3	10	2.6
PH24ST	55	9.3	24	1.1	0	
IRON	27	18.9	6	4.3	4	6.4
ZINC	9	56.7	2	12.8	0	
EPADPADHA	11	46.4	1	25.5	0	
EPADHA	12	42.5	3	8.5	2	12.8
FA_C22_6n3	47	10.9	17	1.5	1	25.5
FA_C22_5n3	6	85.0	1	25.5	1	25.5
FA_C20_5n3	20	25.5	5	5.1	0	
FA_C20_4n6	37	13.8	2	12.8	0	
FA_C20_3n6	35	14.6	9	2.8	4	6.4
FA_C18_2n6	11	46.4	1	25.5	0	
FA_C18_0	23	22.2	7	3.6	0	
FA_C16_0	21	24.3	3	8.5	1	25.5
FA_C14_0	54	9.4	20	1.3	0	
FA_C12_0	31	16.5	3	8.5	1	25.5
FA_C10_0	22	23.2	9	2.8	1	25.5

^a^ = empty cells are not available

**Fig. 2:**
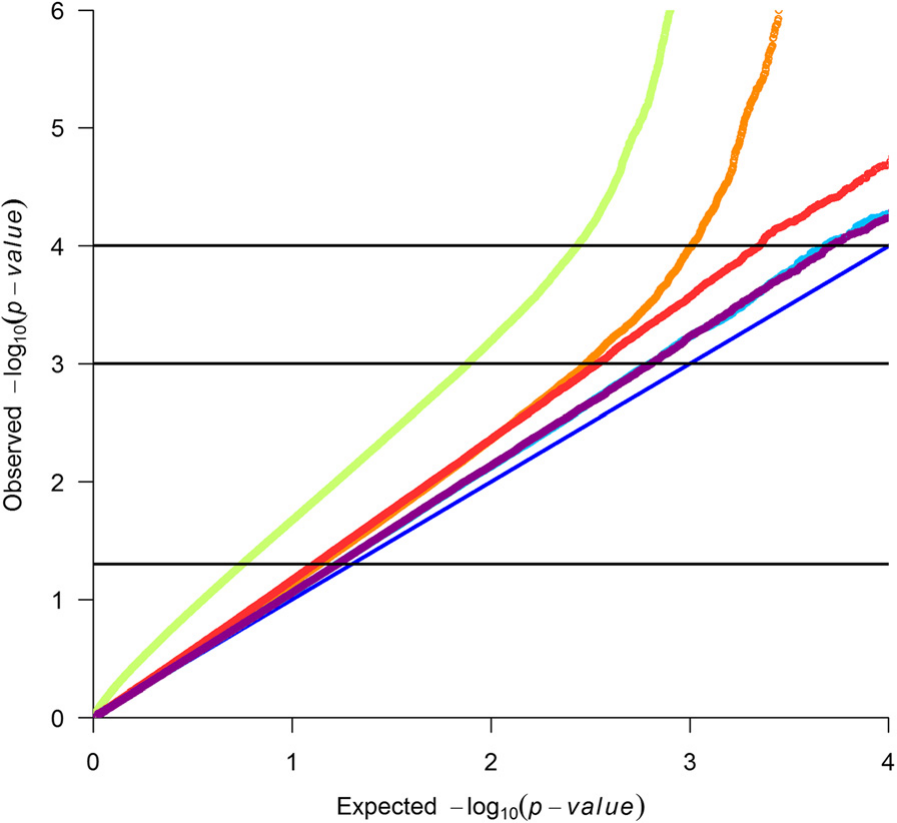
Quantile-quantile plot of *P*-values from single SNP genome wide association study of HGRFAT (*darkorange*), CEMA (*red*), SHEARF1 (*skyblue*), and FA_C22_5n3 (*magenta*), and from multi-trait analysis (*olivegreen1*). Observed and expected *P*-values would fall on the light blue line if there was no association. The top horizontal line is *P* < 0.0001, middle horizontal line is *P* < 0.001, and the bottom horizontal line is *P* < 0.05

Besides FDR, a Q-Q plot can demonstrate the rate of false positives as shown for 4 traits in Fig. 2. The Q-Q line deviates most from expectations in HGRFAT, followed by CEMA, SHEARF1, and FA_C22_5n3, which is consistent with FDR results being lowest in HGRFAT. The deviation of the Q-Q plots from expectation (at low *P* values) is evidence of polygenic inheritance [18], not inflation of the test statistic due to population structure, which has been well captured in our analysis by fitting breed and pedigree (see a model used in single-trait GWAS in materials and methods section). Many significant SNPs were clustered together within narrow regions on chromosome (OAR) 2, 3, 5, 6, 11, 12, 14, 18, 20, and 26. In a number of cases, those SNPs had associations with more than one trait.

### Multi-trait analysis to detect pleiotropy

**Fig. 3:**
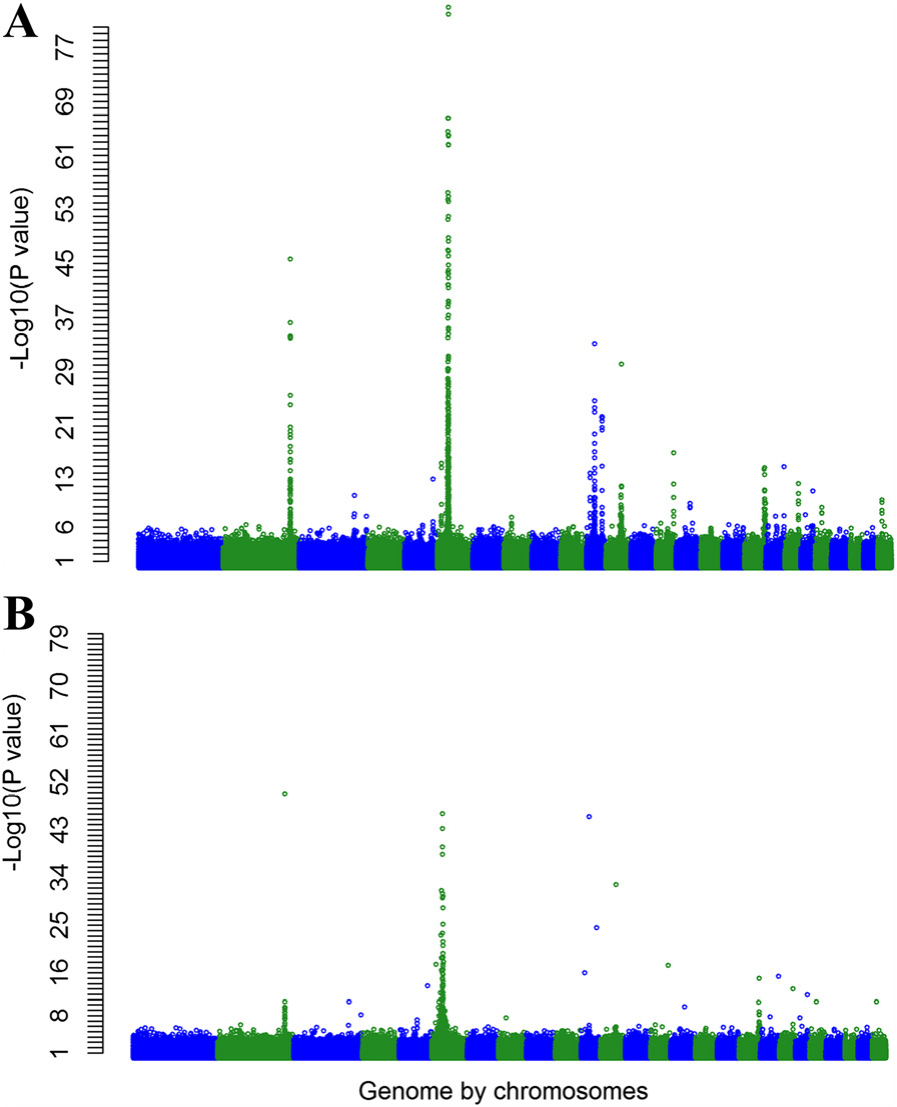
The Manhattan plot showing the –log_10_ (*P*-values) of SNPs of the multi-trait test of the whole genome (except the X chromosome) before (**a**) and after (**b**) fitting 23 lead SNPs in the model

Combining the single trait GWAS in a multi-trait meta-analysis using a method described by [17] resulted in 586 significant SNPs (*P* < 5 × 10^−7^) (Fig. 3a). This corresponded to a FDR of 0.04 %, which was lower than for any individual trait tested in the single-trait GWAS, except LEGBONE.

To avoid testing a large number of closely linked SNPs, only the most significant SNPs (*P* < 10^−5^) within each 1 Mb window (98 SNPs) was selected from the multi-trait analysis in the discovery set (80 % of total of 10,613 animals from 9 different sheep breeds) for validation in an independent set of animals (the remaining 20 % of total animals). For each of 98 significant SNPs (Table 3), we performed a multiple regression analysis in which the SNP genotype is the dependent variable and the 56 phenotypes are the independent variables. This resulted in a linear index of 56 traits that had the maximum correlation with genotypes of one of the corresponding (98) significant SNPs. The association between a SNP and its corresponding linear index was subsequently tested in the validation sample. Out of the 98 SNPs that were found significant in the discovery sample, 35 were significant (*P* < 0.05) in the validation sample and all had an effect in the same direction in the validation sample as in the discovery sample (Table 3). This number validated (35) was higher than for any single trait (PSWT, LEGBONE, DRESS%, and CCFAT; Table 2), showing that multi-trait analysis detected and validated more associations than any single-trait analysis.

**Table 3:** Number of significant SNPs (*P* < 10^−5^) in reference population that were also significant in the validation population

*P* value in validation	No. of SNP	FDR%	%-same
*multi*-*trait*			
0.0001	17	0.05	100
0.001	24	0.3	100
0.01	31	2.2	100
0.05	35	9.5	100
all	98		73
*single*-*trait* (PSWT)			
0.0001	2	0.2	100
0.001	4	0.9	100
0.01	4	9.2	80
0.05	5	34.2	83
all	29		71
*single*-*trait* (LEGBONE)			
0.0001	8	0.06	100
0.001	9	0.5	100
0.01	13	3.4	100
0.05	17	11.2	100
all	48		84
*single*-*trait* (DRESS%)			
0.0001	2	0.19	100
0.001	3	1.23	100
0.01	7	4.67	100
0.05	9	16.4	90
all	31		78
*single*-*trait* (CCFAT)			
0.0001			
0.001	1	2.2	100
0.01	3	6.6	100
0.05	4	22.6	100
all	19		83

%-same = percentage of SNPs, which have an effect in the same direction in both validation and reference sets

**Fig. 4:**
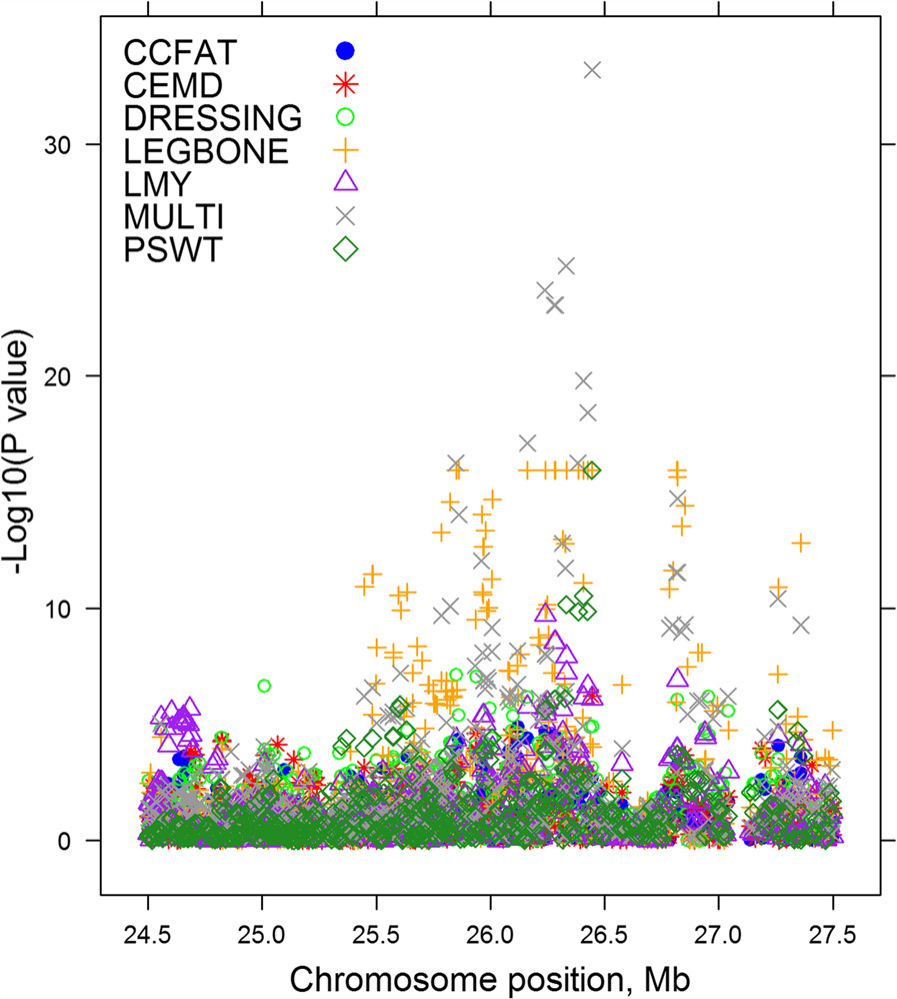
The –log_10_ (*P*-values) of single SNP regressions for 6 traits and multi-trait chi-squared statistic on a region of OAR 11

The multi-trait analysis showed utility to map QTL more precisely. Figure 4 summarises one such case, plotting all significant SNP effects for 6 single trait GWAS and the multi-trait statistic in a region of OAR 11. The multi-trait statistic identifies the SNP at 26,445,930 base pairs as most significant (*P* = 1.32 × 10^−27^), while the 6 separate traits map the QTL between 25,849,323 – 26,445,930 base pairs.

### Conditional analyses accounting for 23 lead SNPs

The multi-trait analyses identified many narrow regions, containing more than one significant SNPs (e.g. on OAR 2, 3, 5, 6, 11, 12, 14, 18, 20, and 26; Fig. 3a). We selected 23 ‘lead SNPs’ (**SNP**_**lead**_) (Table 4), which were significant and not closely linked and therefore presumably tagging 23 independent QTL across the ovine genome. Table 5 lists their t values across all 56 traits. We then tested whether the selected SNP_lead_ were perfectly tagging QTL in their respective regions by rerunning all single trait GWAS, while simultaneously fitting all 23 SNP_lead_ (Table 4). Then the meta-analysis combining all traits was repeated. All 23 lead SNPs remained significant when fitted jointly suggesting that each is likely to tag a different QTL. Fitting the 23 SNPs substantially reduced the number of significant SNPs near the SNP_lead_ (e.g., OAR 14 at 54 Mb and OAR 11 at 13, 26, and 50 Mb; Fig. 3b), showing that the SNP_lead_ is in high linkage disequilibrium with the QTL in the region. However, in some cases (e.g. OAR 6 at 37 Mb), SNPs close to the SNP_lead_ remained significant after fitting all 23 SNP_lead,_ which may indicate that the SNP_lead_ is in imperfect LD with the causal mutation or that there are multiple QTL in the region. In fact, there were still many significant SNPs (*P* < 5 × 10^−7^) scattered throughout the genome after fitting the 23 SNP_lead_ indicating that there are likely to be many more smaller QTL affecting the 56 traits.

**Table 4:** Total number of significant SNPs (*P* < 5 × 10^−7^), their FDR (%), and number of significant SNP on each chromosome (which is in parenthesis) for the 23 linear indexes corresponding to the 23 lead SNPs

Group^a^	SNP order	Linear index^b^ code	Mapped^c^ gene code	Total No. SNP^d^	FDR (%)	chromosome number (number of significant SNPs^e^)
1	1st	OAR16_31.9 Mb	*GHR*	86	0.30	3 (1), 6 (83), 8 (1), 24 (1)
1	2nd	OAR14_34.8 Mb	*LCAT*			
1	3rd	OAR11_26.4 Mb	*SLC16A11*	231	0.11	1 (2), 3 (1), 5 (2), 6 (218), 9 (4), 11 (2), 13 (2)
1	4 th	OAR6_37.5 Mb	*LCORL*	489	0.05	1 (3), 2 (1), 6 (475), 7 (1), 9 (2), 11 (6), 15 (1)
2	5th	OAR26_13.99 Mb	*ACSL*			
2	6th	OAR11_13.3 Mb	*ACACA*	8	3.19	11 (6), 17 (2)
2	7th	OAR11_49.9 Mb	*FASN*	12	2.13	5 (1), 11 (6), 13 (4), 24 (1)
2	8th	OAR6_15.2 Mb	*SNORA70*	14	1.82	6 (12), 11 (1), 13 (1)
3	9th	OAR19_57.1 Mb	*MRPS25*	3	8.50	1 (1), 3 (2)
3	10th	OAR2_219.6 Mb	*PLCD4*	57	0.45	1 (3), 2 (54)
3	11th	OAR3_17.9 Mb	*APOL6*	3	8.50	3 (2), 23 (1)
3	12th	OAR5_93.4 Mb	*CAST*	5	5.10	3 (1), 5 (1), 11 (1), 21 (2)
3	13th	OAR18_64.5 Mb	*MEG8*_*2*	32	0.80	18 (32)
4	14 th	OAR8_25.0 Mb				
4	15th	OAR22_20.3 Mb	*PKD2L1*	11	2.32	1 (1), 6 (2), 18 (8)
4	16th	OAR12_49.6 Mb	*SAMD11*	15	1.70	12 (14), 17 (1)
5	17th	OAR14_54.6 Mb	*GYS1*	10	2.55	2 (6), 4 (1), 10 (1), 18 (2)
5	18th	OAR3_21.9 Mb	*PNPLA3*			
5	19th	OAR21_39.7 Mb	*FADS2*			
5	20th	OAR19_30.8 Mb	*5S*-*rRNA*			
5	21st	OAR20_44.1 Mb	*SMIM13*	9	2.83	20 (9)
5	22nd	OAR21_14.96 Mb				
5	23rd	OAR15_47.5 Mb	*U1*	1	25.51	25 (1)

^a^ = Group of the lead SNPs that were clustered together as shown on Fig. 5

^b^ = 23 linear indexes corresponding to the 23 lead SNPs

^c^ = Genes located within 30 kb from each of lead SNPs excluding *LCORL*, and gene names are in Table 6

^d^ = Total number of significant SNPs which are significantly (*P* < 5 × 10^−7^) associated with each of linear indexes

^e^ = Number of significant SNP on each chromosome is in parentheses

**Table 5:** Effects of 23 lead SNPs on the individual traits (signed values with |t| > 1 are shown)

	Group 1^a^				Group 2^a^				Group 3^a^					Group 4^a^			Group 5^a^						
Lead SNP order^b^	1st	2nd	3rd	4th	5th	6th	7th	8th	9th	10th	11th	12th	13th	14th	15th	16th	17th	18th	19th	20th	21st	22nd	23rd
PSWT	1.8		8.3	6.3			2.4	−1.8				3.7		2.3	2.3							−1.4	2.2
HCWT			5.6	1.6			1.3	−1.4		1.1		3.0	1.0										4.0
LLWT	1.4			1.6					1.1	2.1		3.1	6.5	−1.3	1.6			−1.7					1.3
TOP	2.0		3.4	5.5	−1.2			−1.8		2.9		2.4	3.3							1.2	−1.6	1.4	2.5
RND	2.5		7.2	8.1				−1.7		3.1		1.9		1.4				−1.0				−1.0	2.5
BONE	2.7	2.7	10.9	13.7								2.2		2.6	1.3	−2.2						1.7	1.2
LEGBONE	5.7	2.4	12.3	19.1				−1.3				2.7				−1.6				−1.1	−1.0	2.3	
LMY	3.3	1.2	5.0	8.7		1.2	−1.5			1.9		−1.2	2.9				1.8				−2.5	−2.5	−1.0
DRESS%	−3.6	−3.1	−3.4	−8.7	−2.4			1.2			1.1			−1.6						−1.3	1.5		4.7
DMLOIN	−1.2		−2.6	−6.4							−2.1		−1.3		−2.1		2.7	1.9	1.1				
IMF		−1.7	−1.8	−4.0	2.2				−1.8		−2.0		−5.9		−1.6			2.7	1.3	−1.4	−1.5	1.2	−1.3
LLFAT	−2.5		−4.1	−6.4			2.0	1.8				1.7		1.9		1.1				2.5	3.2		2.7
CFATSCORE	−2.4	1.1	−4.1	−4.0			1.1	1.2		1.4	−1.5	−1.4									2.0		
CCFAT	−2.5	−2.9	−3.1	−9.2			3.0	1.4			−2.1	1.9	−1.7	1.2	−1.5		−1.7				1.3		
HGRFAT	−3.4	−1.9	−7.1	−9.3			1.8		2.2		−2.0	1.6		1.3			−1.5			1.9	1.5	1.5	1.0
CFAT5	−2.2	−2.2	−4.6	−4.6		−2.0	2.0		1.4			1.9	−1.2				−1.5			2.9			
CEMW	2.1		1.2	5.4	1.1	1.2							1.1	−2.2								−1.9	2.3
CEMD	−2.9	−1.8	−5.0	−3.8	1.4				3.7			1.5		−2.5		1.6				1.6	−1.5		
CEMA	−1.0	−1.9	−3.4		1.1				2.7			1.6	2.0	−3.5		1.0					−1.6		1.9
SHEARF5	2.5	1.2	3.3	3.7	1.2	−1.0			−3.4	−2.6		8.0	5.6	1.2				−1.1		1.8	1.2	−4.5	
SHEARF1	2.7	1.8	1.1	1.8	1.4		−1.2		−2.4	−1.3		5.5	3.3		−1.5				−1.3	2.6		−1.3	
MYOGLOBIN		2.4	−2.3	−3.6		−1.3			5.4	5.5	6.3	2.1	2.0		2.1	1.4	−1.9				−1.2		
GLYCOGEN		2.0				−1.9			1.5	7.2	1.0					1.0	8.8						−1.0
ICDHACTIVITY	−1.2		−1.5	1.4		−1.4				5.4			−1.8			−6.9			−1.8	1.4		−1.0	−1.4
RCL4	−1.1		1.4	2.3			1.3		−4.9	−4.3	−5.1	−1.5	−3.0	−1.5	−2.5	−3.4			1.6				2.3
RCL3	−2.1		2.0	2.2					−4.0	−5.5	−4.6	−2.5	−3.7	−2.3	−2.2	−3.1			2.0				2.5
RCL2	−1.4		1.6	2.8					−3.7	−5.3	−4.2	−2.9	−3.7	−2.7	−2.5	−3.5		−1.5	1.3	1.2			1.7
RCL1			1.2	1.8					−3.8	−2.3	−3.8		−1.6		−2.7	−1.1	3.7	1.1	1.9	1.9		1.3	1.2
RCb4			1.2	−1.3						−2.6	1.1					2.9			1.6		1.6	−1.1	
RCb3		−1.7		−2.0								1.7	1.0	1.5	1.2	1.8		−2.8					
RCb2				−2.8	−1.2		1.1	−1.1				2.5		1.5	2.0	2.6	2.2						
RCb1			−1.7	−2.6				−2.0	−1.8	5.0		1.9				3.6	3.2		1.4		1.3		
RCa4		−1.6		−3.3				−1.6			4.1	3.9	2.1	1.3	1.4	7.4	4.1	1.5	2.1		2.4	−1.5	−1.1
RCa3	1.1	−2.7	−1.1	−3.9		1.2		−1.7		2.0	3.7	5.6	2.6	2.0		7.5	4.7		1.6		1.6		
RCa2	1.2	−2.4	−1.7	−4.1	−1.3			−1.6		3.6	3.2	5.3	2.3	1.6		7.2	6.0	1.4	1.5		1.3	−1.1	
RCa1		−1.1	−3.3	−4.1				−1.8	2.0	6.7	2.8	3.2	2.0	1.5		6.6	3.0				1.4		−2.6
CFb	−1.2			−3.1			1.3		−2.9	1.3		−1.2		2.4		2.9						3.1	
CFa			−1.6	−3.9	−2.4					6.9	3.9	−1.0		3.4		4.3	−1.4	−1.2				1.9	
CFL	−1.2	1.0	1.3	1.3					−5.9	−2.4	−6.1		−3.7			−2.3	1.7		2.5	−1.1			
PH24LL			2.5	3.0					−2.1	−11.1			−2.5			−1.1	−4.9			−3.1		−1.9	1.6
PH24ST				−1.4					−2.0	−9.0		−1.1			1.3		−5.6		−1.2			−1.1	−2.5
IRON			−2.8	−1.5			1.4		5.2	6.2	3.7		1.4	1.5			−1.9		−1.3	1.1	1.2	−2.7	−3.5
ZINC	1.3			−2.3					−3.7				−2.3						1.1		−1.7	−1.9	
EPADPADHA		1.1		−1.8		1.1	−1.3		2.5	3.3	−1.1				−1.2	−7.6	−2.2	3.6				−1.3	1.3
EPADHA		1.0				1.2			1.9	4.3			1.5	−1.3		−7.5	−2.1	2.6		1.6	1.9	−1.2	−1.8
FA_C22_6n3	2.5	1.1	−1.4		2.8	1.0		−1.8	2.4	3.6				−1.3	−1.3	−6.8	−1.0		1.5	2.6	6.8	−1.6	
FA_C22_5n3				−1.5					2.8	1.0	−1.4	−1.1		1.0	−1.3	−5.8		3.5			−2.7		
FA_C20_5n3						1.1			2.4	4.1	−1.6		2.2		−1.1	−8.2		3.3					2.4
FA_C20_4n6	2.0			1.3	1.5	1.3		−1.4	1.9	5.4	−2.3	1.2	1.1			−4.6	−2.9	−1.6	3.1				
FA_C20_3n6	2.1	−1.4			−1.2	2.6		−1.2	2.2	2.8	−2.0		2.1			−5.7		2.4	8.2				1.3
FA_C18_2n6					2.7					3.7					−2.0	−6.1			−1.8		−2.2		
FA_C18_0		−1.3	2.3	2.5		−2.5	−1.6	−1.8	−1.0			−2.1	−3.0	−2.7	5.5	1.4				1.2			1.2
FA_C16_0	−1.8					3.6	4.9	6.0		−1.6		−1.1	−1.3	−1.6	1.2			−2.0		1.2	−1.0	−1.1	
FA_C14_0	−2.7			−2.7		4.9	8.4	3.7		−2.9			−1.2										
FA_C12_0	−1.7	1.0		−3.6	−4.5	2.2	3.3	3.0		−3.3				−1.7				2.9		2.3			−1.9
FA_C10_0		1.5			−3.3	7.2	3.8	3.0	−2.1	−3.0	−1.2		−1.1		1.3			1.2		2.4		−2.0	

^a^Group of the lead SNPs that were clustered together as shown on Fig. 5, ^b^This SNP order refers SNPs, which are given on Table 4

### Cluster analysis to find QTL with a similar pattern of effects across traits

**Fig. 5:**
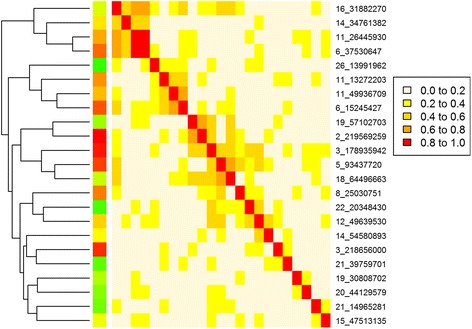
Correlation matrix between the 23 lead SNPs calculated from SNP effects on 56 traits

The correlation of effects across all 56 traits was calculated for all pairs of SNP_lead_ (Fig. 5). While the correlation of effects for the SNP_lead_ 3 (OAR 6) and the SNP_lead_ 4 (OAR 11) was more than 0.8, most correlations were moderate to low. A moderate to low correlation suggests QTL with different patterns of effects across traits, however, sampling errors in estimating SNP effects also reduce the absolute value of the correlation. If two QTL affect the same physiological pathway one might expect them to have the same pattern of effects and, hence, a higher correlation. Cluster analysis of the 23 SNP_lead_ (Table 4) based on the correlation matrix divided them into 5 loosely defined groups (Fig. 5), which shared patterns of effects across traits (Table 5).

Group 1 consisted of 4 SNP_lead_ on OAR 16, 14, 11 and 6 (Table 4 and Fig. 5). This group clustered as an outer branch (Fig. 5), indicating that this group of SNPs was distinct from the other 4 groups. Table 5 shows that these 4 SNP_lead_ increased carcass and skeletal weights and lean meat yield and decreased dressing percentage, fatness, and muscling. They could be described as changing mature (skeletal or carcass) size.

Group 2 consisted of 4 SNP_lead_ (Table 4 and Fig. 5). These SNP_lead_ had an allele that increased the concentration of saturated FA with carbon chain of C16, C14, C12, and C10 (palmitic, myristic, lauric, and capric acids, respectively) and decreased stearic acid (C18:0) and/or some unsaturated FA (Table 5). There was also a tendency for the allele that increased saturated FA composition to also increase fatness (Table 5). They could be described as changing fat composition.

Group 3 consisted of 5 SNP_lead_ that influenced meat retail colour (by increasing redness of meat (RCa^*^) and decreasing lightness of meat (RCl^*^)) and increased myoglobin and wet iron content in muscle. However, on other traits, they separated into two subgroups (Table 4 and Fig. 5). One sub-group consisted of 2 SNPs (9th and 10th SNP_lead_) that increased meat tenderness (i.e., decreased shear force), eye muscle area and eye muscle depth, glycogen, isocitrate dehydrogenase (ICDH) activity, and polyunsaturated FA (omega-3 and -6) level and decreased meat pH level (Table 5). The other sub-group contained 3 SNPs (11th, 12th, and 13th SNP_lead_), and 2 of them (12th and 13th SNP_lead_) had decreased meat tenderness and increased top side, loin weight and eye muscular area. Additionally, the 13th SNP_lead_ (OAR18_64.5 Mb) decreased intramuscular fatness and increased leanness. They could be described as influencing meat colour and eating quality.

Group 4 consisted of 3 SNP_lead_ (Table 4). Correlations between these SNP_lead_ were only moderate and they were also moderately correlated with some SNP_lead_ from group 3 (Fig. 5). This group tended to influence meat colour and polyunsaturated (omega-3 and -6) FA levels (Table 5). Additionally, the 14th SNP_lead_ decreased muscling and the 16th SNP_lead_ significantly decreased ICDH activity and omega-3 and -6 FA levels. They could be described as affecting meat colour and FA composition.

Group 5 consisted of 7 SNPs, which were less correlated and do not form a consistent group (Fig. 5). The 17th SNP (OAR14_54.6 Mb) increased glycogen content and meat redness (increased FCa*), and decreased ultimate pH (pH24) (Table 5). This was similar to the 10th and 9th SNP_lead_ in Group 3, but it did not affect tenderness. The 23rd SNP_lead_ (OAR15_47.5 Mb) increased hot carcass weight and dressing percentage and decreased iron content. Each of the other 5 SNPs (18th-22nd SNP_lead_) in this group had a significant effect on specific traits including FA composition or fatness or tenderness.

### Searching for more QTL in the same pathway using linear indices of SNP_lead_

Genes that operate in the same pathway might be expected to show the same pattern of pleiotropic effects. We wanted to harness the power of our multi-trait analysis to add additional QTL to the 5 broad functional groups. For each of the 23 lead SNPs, we used the same linear index as was used previously to validate the SNP effects. That is, we calculated the linear combination of the 56 traits that was most highly correlated with the genotypes at each of the SNP_lead_ [19]. Then we performed new GWAS using the linear index as if it was a new trait. All SNP_lead_ were also fitted simultaneously in the GWAS, as we were primarily interested in finding additional QTL to those captured by the SNP_lead_. This process added a total of 687 significant SNPs (*P* < 5 × 10^−7^) that were assigned to the same group as the SNP_lead_ whose linear index was used as the phenotype (Table 4). Usually this procedure identified a set of closely linked SNPs, presumably indicating a single QTL.

### Identifying plausible candidates

We searched for genes within genomic regions of 30 kb up and downstream from each of 687 SNPs from linear index GWAS and the 23 SNP_lead_. If there were multiple significant SNPs within a 60 kb window only the most significant SNP was taken forward. The closest gene was chosen as a likely candidate. In one exception (SNP_lead_ 4 at OAR6_37.5 Mb), we expanded the 60 kb range as the nearest gene (*LCORL*) was 78 kb away (Additional file 1: Figure S1a). This identified 71 SNPs in or close to potential candidate genes (Table 6). Table 6 lists these genes in genome position order.

**Table 6:** List of plausible candidate genes

Group	Lead SNP	OAR	POS	Gene code	Gene name	Comments
Group1	1st	16	31882270	*GHR*	Growth hormone receptor precursor	Lead SNP
Group1	1st	8	73201627	*UST*	uronyl-2-sulfotransferase	Linear Index SNP
Group1	2nd	14	34761382	*LCAT*	lecithin-cholesterol acyltransferase	Lead SNP
Group1	3rd	11	26445930	*SLC16A13* (*TP53* ^*a*^)	Solute carrier family 16 member	Lead SNP
Group1	3rd	13	700413	*PLCB1*	Phosphoinositide phospholipase C	Linear Index SNP
Group1	3rd	5	28332019	*SNX24*	Sorting nexin-24	Linear Index SNP
Group1	3rd	6	40133729	*PACRGL*	PARK2 Co-Regulated-Like	Linear Index SNP
Group1	3rd	6	44672729	*PI4K2B*	phosphatidylinositol 4-kinase type 2 beta	Linear Index SNP
Group1	4 th	6	43309694	*PPARGC1A*	Peroxisome proliferator-activated receptor gamma coactivator 1-alpha	Linear Index SNP
Group1	4 th	11	26415211	*ALOX12*	12-lipoxygenase Fragment	Linear Index SNP
Group1	4 th	11	28366019	*STX8*	Syntaxin 8	Linear Index SNP
Group1	4 th	1	171579973	*MYH15*	myosin, heavy chain 15	Linear Index SNP
Group1	4 th	6	13255764	*ALPK1*	alpha-kinase 1	Linear Index SNP
Group1	4 th	6	19164907	*TBCK*	TBC1 domain containing kinase	Linear Index SNP
Group1	4 th	6	23695577	*PPP3CA*	protein phosphatase 3, Catalytic Subunit, Alpha Isozyme	Linear Index SNP
Group1	4 th	6	26074029	*RAP1GDS1*	Rap1 GTPase-GDP dissociation stimulator 1	Linear Index SNP
Group1	4 th	6	29441012	*BMPR1B*	bone morphogenetic protein receptor, type IB	Linear Index SNP
Group1	4 th	6	36811936	*MEPE*	Matrix Extracellular Phosphoglycoprotein	Linear Index SNP
Group1	4 th	6	37237578	*NCAPG*	non-SMC condensin I complex, subunit G	Linear Index SNP
Group1	4 th	6	37530647	*LCORL*	Ligand Dependent Nuclear Receptor Corepressor-Like	Lead SNP
Group1	4 th	6	55607047	*ARAP2*	ArfGAP with RhoGAP domain, ankyrin repeat and PH domain 2	Linear Index SNP
Group1	4 t	6	67191744	*FRYL*	FRY-like	Linear Index SNP
Group1	4th	9	36164331	*PLAG1*	pleiomorphic adenoma gene 1	Linear Index SNP
Group2	5th	26	13991962	*ACSL1*	acyl-CoA synthetase long-chain family member 1	Lead SNP
Group2	6th	11	13223903	*ACACA*	acetyl-CoA carboxylase alpha	Linear Index SNP
Group2	6th	11	13272203			Lead SNP
Group2	6th	11	13472077	*SYNRG*	synergin, gamma	Linear Index SNP
Group2	7th	11	49936709	*FASN*	Fatty acid synthase Fragment	Lead SNP
Group2	7th	13	71732382	*SGK2*	serum/glucocorticoid regulated kinase 2	Linear Index SNP
Group2	7th	24	33535204	*MLXIPL*	MLX interacting protein-like	Linear Index SNP
Group2	7th	5	4503837	*ISYNA1*	Inositol-3-phosphate synthase 1	Linear Index SNP
Group2	8th	6	15245427	*SNORA70*	small nucleolar RNA, H/ACA box 70	Lead SNP
Group2	8th	6	15303638	*ELOVL6*	ELOVL fatty acid elongase 6	Linear Index SNP
Group2	8th	6	97914579	*AGPAT9*	1-acylglycerol-3-phosphate O-acyltransferase 9	Linear Index SNP
Group3	9th	19	57102703	*MRPS25*	28S ribosomal protein S25, mitochondrial	Lead SNP
Group3	9th	3	178861487	*MB*	Myoglobin	Linear Index SNP
Group3	10th	1	31018467	*PRKAA2*	protein kinase, AMP-activated, alpha 2 catalytic subunit	Linear Index SNP
Group3	10th	2	212089089	*ERBB4*	v-erb-b2 avian erythroblastic leukemia viral oncogene homolog 4	Linear Index SNP
Group3	10th	2	219569259	*PLCD4*	1-phosphatidylinositol-4,5-bisphosphate phosphodiesterase delta-4	Lead SNP
Group3	10th	2	219741728	*CYP27A1*	cytochrome P450, family 27, subfamily A, polypeptide 1	Linear Index SNP
Group3	11th	23	5077077	*NETO1*	neuropilin (NRP) and tolloid (TLL)-like 1	Linear Index SNP
Group3	11th	3	178935942	*APOL6*	apolipoprotein L, 6	Lead SNP
Group3	12th	11	19757132	*PROCA1*	Protein PROCA1	Linear Index SNP
Group3	12th	21	42744428	*CAPN1*	Calpain-1 catalytic subunit	Linear Index SNP
Group3	12th	3	193152752	*ABCC9*	ATP-binding cassette, sub-family C (CFTR/MRP), member 9	Linear Index SNP
Group3	12th	5	93437720	*CAST*	Calpastatin	Lead SNP
Group3	13th	18	63919438	*WARS*	Tryptophanyl-tRNA synthetase, cytoplasmic	Linear Index SNP
Group3	13th	18	64095685	*BEGAIN*	brain-enriched guanylate kinase-associated	Linear Index SNP
Group3	13th	18	64349803	*DLK1*	delta-like 1 homolog	Linear Index SNP
Group3	13th	18	64452243	*MEG3*_*2*	maternally expressed 3	Linear Index SNP
Group3	13th	18	64496663	*MEG8*_*2*	maternally expressed 8	Lead SNP
Group3	13th	18	66755669	*MARK3*	MAP/microtubule affinity-regulating kinase 3	Linear Index SNP
Group4	14th	8	25030751	(*LAMA4*)	laminin, alpha 4	Lead SNP
Group4	15th	18	6026853	*MEF2A*	Myocyte-specific enhancer factor 2A	Linear Index SNP
Group4	15th	1	373512	*FARP2*	FERM, RhoGEF and pleckstrin domain protein 2	Linear Index SNP
Group4	15th	22	20348430	*PKD2L1* (*SCD* ^*b*^)	polycystic kidney disease 2-like 1	Lead SNP
Group4	16th	12	49339905	*B3GALT6*	UDP-Gal-betaGal beta 1,3-galactosyltransferase polypeptide 6	Linear Index SNP
Group4	16th	12	49639530	*SAMD11*	sterile alpha motif domain containing 11	Lead SNP
Group4	16th	17	63396022	*MYO1H*	myosin IH	Linear Index SNP
Group4	16th	12	49270130	*GLTPD1*	glycolipid transfer protein domain containing 1	Linear Index SNP
Group5	17th	14	54580893	*GYS1*	Glycogen synthase, muscle	Lead SNP
Group5	17th	4	54562436	*PPP1R3A*	protein phosphatase 1, regulatory (inhibitor) subunit 3A	Linear Index SNP
Group5	18th	3	218656000	*PNPLA3*	patatin-like phospholipase domain containing 3	Lead SNP
Group5	19th	21	39759701	*FADS2*	Fatty acid desaturase 2	Lead SNP
Group5	20th	19	30808702	*5S*_*rRNA*	RNA, 5S ribosomal	Lead SNP
Group5	21th	20	44129579	*SMIM13*	small integral membrane protein 13	Lead SNP
Group5	21th	20	44237093	*ELOVL2*	ELOVL fatty acid elongase 2	Linear Index SNP
Group5	22th	21	14965281			Lead SNP
Group5	23th	15	47513135	*U1*	RNA, variant U1	Lead SNP
Group5	23th	25	29372500	*USP54*	ubiquitin specific peptidase 54	Linear Index SNP

^a^ *TP53* and ^*b*^ *SCD* genes which were mapped near this region could be the plausible candidates

**Fig. 6:**
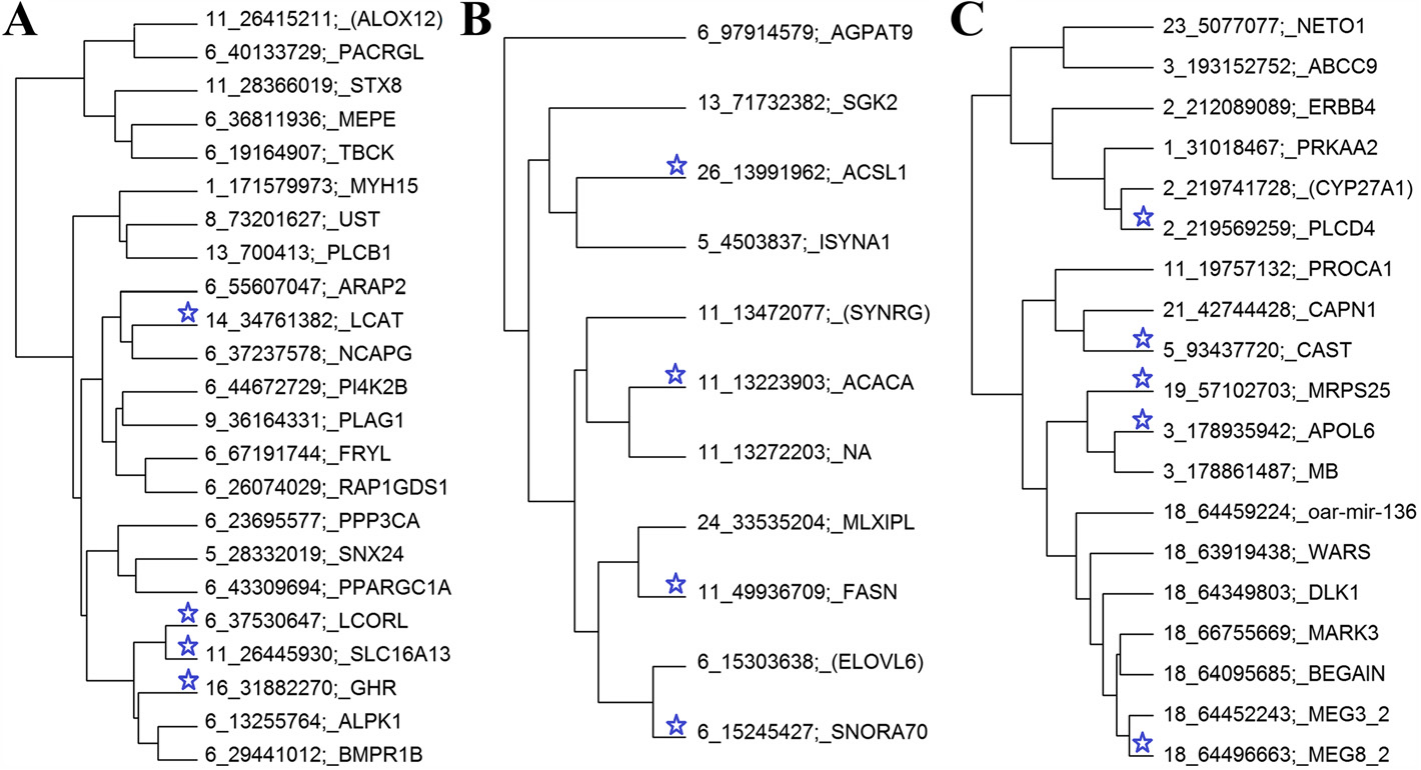
Dendrogram drawn based on correlation matrix between the effects of the lead SNPs and their linear index SNPs within each group: **a** Group 1 SNPs (chromosome and position in base pair) along with their annotated gene names; **b** Group 2 SNPs; and **c** Group 3 SNPs. Lead SNPs within each group are highlighted with blue stars; Genes (in *brackets*) are the alternative most likely putative candidates within the regions

In Group 1, 23 SNPs (including 4 SNP_lead_) were annotated to possible candidate genes (Fig. 6a and Table 6). The function of *GHR* makes this gene a plausible member of a group affecting mature size and exogenous administration of growth hormone leads to increased growth and decreased fatness which is the phenotype of SNP_lead_ 1 near the *GHR* gene. However, in general, the 23 genes in this group do not share an obvious biological function although 4 (*GHR*, *LCORL*, *LCAT*, *PLAG1*) genes have been associated with a similar phenotype in other species as discussed below. *NCAPG* (non-SMC condensing I complex, subunit G) is closely linked to *LCORL*, so there may be only one QTL in this region [9, 20, 21]. Not surprisingly, the effects of SNPs near *LCORL* and *NCAPG* are highly correlated. The SNPs tagging *LCORL*, S*LC16A13*, *GHR*, *FRYL*, and *BMPR1B* were clustered together as one group within Group 1 (Fig. 6a) and the effects of those SNPs were highly correlated (*r* > 0.6).

The 4 SNP_lead_ in Group 2 were expanded with 7 additional SNPs from the linear index GWAS and re-clustered within the group (Fig. 6b). The 11 SNPs tag only 8 different chromosomal regions and in 7 of these there is a gene directly involved in FA synthesis or fat synthesis (*FASN*, *MLXIPL*, *EVOLV6*, *ACACA*, *SYNRG*, *ACSL1*, *ISYNA1*, *SGK2*, and *AGPAT9*) (Fig. 6b). Although the closest gene to SNP_lead_ 6 is *SNORA70*, the analysis of the linear index derived from SNP_lead_ 3, identified a significant SNP only 60 kb away whose nearest gene is *ELOVL6*, which is a far more plausible candidate. *ACSL1*, *ACACA* (said to be the rate limiting step), *FASN* and *EVOLV6* code for enzymes used in fatty acid synthesis and the SNPs near them all have an allele that increases the proportion of C10 to C16 saturated FAs (Table 5). Usually FA synthesis does not proceed to chains longer than C16. It is therefore understandable that the allele that increases C10 to C16 FAs tends to increase total fatness (Table 5). *AGPAT9* encodes an enzyme used in triglyceride synthesis. The *MLXIPL* protein activates carbohydrate response element motifs in the promoters of triglyceride synthesis genes. GO and KEGG analysis in STRING (functional protein association network program) [22] confirms this functional similarity between the genes near group 2 SNPs (Additional file 2: Figure S2). For example, according to KEGG and GO terms, 2 proteins (FASN and ACACA) were involved together in FA biosynthesis (Bonferroni *P* = 3.8 × 10^−4^), 5 proteins (FASN, ACACA, ACSL1 (or FACL2), AGPAT9, and ISYNA1) in metabolic pathways (Bonferroni *P* = 7.6 × 10^−3^), and 4 proteins (FASN, ELOVL6, ACSL1, and ISYNA1) in lipid biosynthesis process (Bonferroni *P* = 3.8 × 10^−2^).

Group 3 (Fig. 6c) consisted of 19 SNPs including 5 SNP_lead_ that were assigned in this group previously. We called Group 3 SNPs “meat colour” SNPs because this is the most consistent feature of the group. The allele that made the meat redder and darker also tended to increase myoglobin and iron content, decrease pH and increase muscling. However, in other respects the SNPs in group 3 differ in their phenotypic effects. The 9th and 10th SNP_lead_ decrease shear force while the 12th and 13th SNP_lead_ increase it. Considering the differences in phenotype and in the function of the candidate genes, there may be no single physiological process that is common to all SNPs in group 3.

**Fig. 7:**
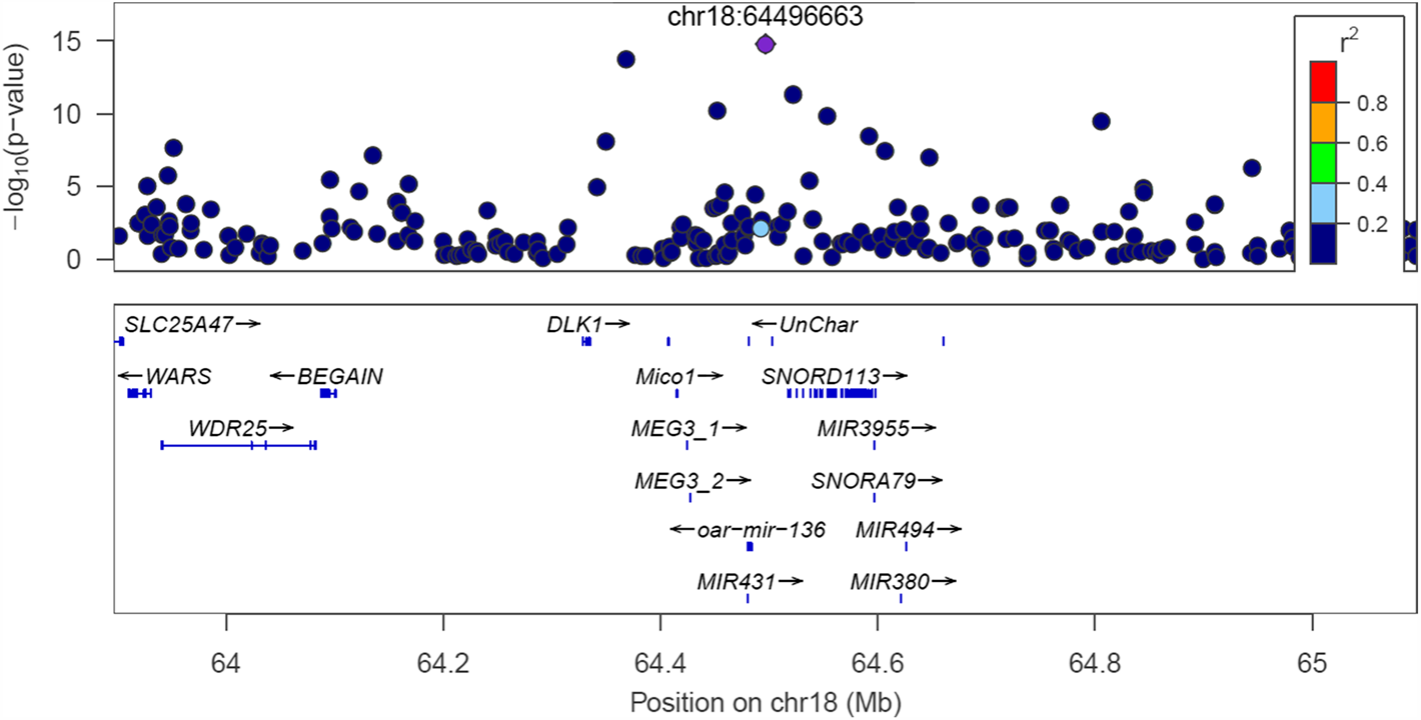
The –log_10_ (*P*-values) of SNP effects from the multi-trait test results for OAR18_64.5 Mb, where not all genes in this region are shown: The lead SNP is shown by a purple diamond in each plot (labelled with chromosome and position, Mb) and the LD between this variant and all others is colour coded

One group of SNPs were found within OAR 18 63.3-65.6 Mb near *MEG3* (*or GTL2*), *MEG8*, *DLK1*, *oar*-*mir*-*136* (or *PEG11*), *BEGAIN*, and *WARS* (Fig. 7). This SNP increases muscling and shear force which is the phenotype of the callipyge mutation which maps to the same region [15, 16]. The callipyge mutation is not known to occur in Australian sheep but the Carwell mutation has a similar, but less dramatic phenotype, and maps to the same region [16, 23] so it is likely that the SNP_lead_ 13 is tagging the Carwell mutation. The SNP_lead_ 12 tags *CAST* and the linear index derived from this SNP was significant for a SNP tagging *CAPN1*. Both *CAST* and *CAPN1* have also been linked to tenderness in cattle [24–26]. The effects of the SNP_lead_ 12 (tagging *CAST*) and 13 (tagging Carwell) were highly correlated (Fig. 5). Although these genes do not have similar functions, both decrease muscle protein turnover which may help to explain why they both increase muscling and decrease tenderness. The SNP_lead_ 11 (OAR3_17.8 Mb) was mapped near the genes *APOL6* (apolipoprotein L6) and *MB* (myoglobin) and was strongly associated with meat myoglobin content and also FA composition.

Group 4 and 5 consist of a total of 18 SNPs which do not cluster tightly and do not show any obvious common mechanism although individual candidate genes do have a function closely related to the phenotypic effects of the SNP tagging them. The SNP_lead_ 17 at OAR14_54.5 Mb of Group 5 has a strong association with muscle glycogen content but not with ICDH activity (Table 5) and mapped within the region of the gene *GYS1* (glycogen synthase). The SNP_lead_ 19 (OAR21_39.7 Mb) and 18 (OAR3_21.8 Mb) mapped near genes *FADS2* (a component of a lipid metabolic pathway that catalyzes biosynthesis of highly unsaturated FA) and *PNPLA3* (which is involved in both triacylglycerol lipase and acylglycerol O-acyltransferase activities), respectively. In this study, these SNPs were strongly associated with poly-unsaturated FA concentration. Similarly, the linear index of the 21th SNP_lead_ (OAR20_44.1 Mb) was associated with a SNP only 100 kb from the SNP_lead_ and close to the gene *ELOVL2* (Table 6). This SNP_lead_ had a strong positive effect (*t* = 6.8) on docosapentaenoic acid (DHA or FA_C22_6n3), but not with eicosapentaenoic acid (EPA or FA_C20_5n3) (Table 5). *ELOVL2* adds 2 carbons to polyunsaturated long chain FAs so it is a logical candidate for the effect of both SNPs. The same pattern of effects of a SNP in *ELOVL2* was observed in the human GOLDN study [19]. Furthermore, *SCD* (delta-9 desaturase), which is involved in fatty acid biosynthesis, primarily the synthesis of oleic acid (FA_C18_1n-9) by desaturation of stearic acid (FA_C18_0), was located just in 45 kb away from the 15th SNP_lead_ (OAR22_20.3 Mb) (Additional file 1: Figure S1b). So, *SCD* may be a plausible candidate gene for this region. We investigated further if there were any significant SNPs near or within *SCD*, which are in LD with the SNP_lead_ OAR22_20.3 Mb. There were 3 significant SNPs (*P* < 3.1 × 10^−4^), located 8.5 kb-22.5 kb upstream to the *SCD* gene and all 3 were in LD (*r*^2^ = 0.16-0.67) with this SNP_lead_ (Additional file 1: Figure S1b).

### Do these candidate genes affect similar phenotypes in other species?

#### Comparison with cattle

Of the genes in Table 6 (i.e. near 23 SNP_lead_ or SNPs highly significantly associated with linear indices derived from the SNP_lead_), 3 (*LCAT*, *FADS* and *PLAG1*) were also highly significant and had similar effects in a multi-trait analysis of beef cattle [27]. Of the other genes found by [27], in this study we found 6 SNPs (*P* < 10^−3^) within the region of gene *LEPR*, 1 SNP (*P* = 1.5 × 10^−3^) within *HMGA2*, 1 SNP (*P* = 4.1 × 10^−5^) within *PLIN3*, and 1 SNP (*P* = 8.8 × 10^−6^) within *PACRG*. Saatchi et al. [9] also found QTL regions harbouring genes associated with growth including *PLAG1*, *LCORL*, *NCAPG*, and *HMGA2* in their GWAS in 18,000 animals from 10 US beef cattle breeds.

#### Comparison with human GWAS

We also investigated the overlap of our QTL with genes associated with height, BMI, waist to hip ratio, and obesity in humans, which are well documented and have been validated in multiple studies. We selected the BMI and BMI-related gene lists reported in 3 recent human meta-analyses [2, 27–29]. In total, 229 unique genes from these 3 studies (96 [2], 58 [27], and 78 [28, 29] genes) were tested. 184 of these genes could be mapped to positions on the sheep reference genome (OAR 3.1; [30]). We detected 137 SNPs that were significant (*P* < 10^−3^) in our meta-analysis and that mapped to 55 of the 184 human genes (Additional file 3: Table S1). In 1,000 permutations of the data we did not observe a case with these many SNPs and genes overlapping between the human and sheep results. These 55 genes included *PPARGC1A* (identified from the 4 th SNP_lead_ linear index) and *PLCD4* (tagged by the 10th SNP_lead_), and the two genes identified by the linear index of the 10th SNP_lead_, *CYP27A1* and *ERBB4. PLCD4*, *CYP27A1 and ERBB4* map near each other so it is uncertain how many causal mutations are involved (Additional file 2: Table S1). We also detected 9 SNPs with *P* values from 8 × 10^−4^ to 9.7 × 10^−18^ near the gene *FTL* (ferritin light polypeptide), which is known to be strongly associated with human obesity and carriers of the risk allele reported to have increased appetite [31]. The position of *FTL* gene in ovine genome was partially overlapped with *GYS1* gene.

We also evaluated the overlap between the sheep GWAS results described above and GWAS for human height. Out of 697 SNPs annotated to 604 genes associated with human height [6], 494 genes mapped to the sheep reference genome and 287 SNPs which were significant (*P* < 10^−3^) in our meta-analysis mapped to 118 of these 494 human genes. Again in 1,000 permutations we did not observe a case with this much overlap between sheep and human lists. Out of these 287 SNPs, 73 SNPs at *P* < 10^−5^ were mapped within or near 11 genes which was presented in Additional file 3: Table S1. These 11 genes [6] included *LCORL* (tagged by the 4 th SNP_lead_ of group 1), *GHR* (tagged by the 1st SNP_lead_ of group 1), and *MARK3* (tagged by SNP associated linear index of the 13th SNP_lead_ of group 3). Gene *TP53* from [6] (Additional file 3: Table S1) was located on OAR11 at 26.9 Mb, which is close to gene *SLC16A11* that was near the 3rd SNP_lead_ in group 1, so *TP53* might be the plausible candidate gene in this region.

## Discussion

### Multi-trait analysis increases power

We have demonstrated that for a wide range of carcass and fat composition traits, a multi-trait GWAS strategy (combining single-trait GWAS in a meta-analysis) detected and validated more QTL than simple single-trait GWAS. The FDR was low for the majority of traits studied (Table 2 and Fig. 2). The Q-Q plot deviated from expectation at very low values of –log_10_ (*P* value) for the multi-trait analysis but, since we fitted a breed and a polygenic effect in the model, this deviation is not likely due to uncontrolled population structure. This type of deviation from expectation is observed if many loci cause genetic variation for a particular trait [18]. The increase in power from the multi-trait analysis was possibly due to the fact that all traits measure aspects of muscle and fat growth and thus share some underlying biological mechanisms.

### Candidate genes

The gene closest to the most significant SNP is not necessarily the gene responsible for the effect on phenotype. However, in some cases the candidate genes in Tables 4 and 6 are likely to be correct based on two sources of evidence. In some cases the known function of the candidate gene fits the observed phenotype very well. For instance, the gene glycogen synthase is a good candidate for affecting muscle glycogen concentration. In other cases, the same gene has been reported to affect the same trait in another species. For instance, *LCAT* affects mature size in cattle even though a causal relationship is not apparent from a metabolic biochemsitry perspective [17]. Both types of evidence support some candidates such as *CAST*, *CAPN1*, and *GHR*. However, in some cases we identified more than one closely linked genes as candidates. The causal gene could be any one of these or, in some cases, multiple causal variants may exist in the same region. For instance, the effects of SNP_lead_ 11 involve both myoglobin content and FA composition which suggest that both *MB* and *APOL6* play a role.

### Do detailed phenotypes help to identify the causal gene?

In some cases they do. For instance, the group 2 genes have a large effect on FA composition and a small effect on fatness. Without the FA data, their effect on fatness would have been overlooked. Similarly, *GYS1* has a large effect on muscle glycogen and pH and a smaller effect on other traits. These cases might be described as measuring a phenotype which is close to the primary action of the gene. For instance, *GYS1* codes for the enzyme that synthesises glycogen. By contrast, traits such as body weight are far removed from the direct action of any one gene. When a gene codes for an enzyme it is easier to specify a phenotype close to the primary action of the gene (e.g. the amount of the product) than in many other cases.

### Do QTL with similar patterns of effects across traits tag genes in the same pathway?

We used two methods to identify SNPs with a similar pattern of associations across traits–clustering the lead SNPs and using a linear index designed for one SNP to find others with similar effects. The genes that cluster together in group 2 belong to the fat synthesis pathway but the candidate genes in other groups do not share an obvious pathway or mechanism. Using the linear index derived from one SNP_lead_ did, in some cases, find other genes in the same pathway. For instance, the linear index that best predicts the *CAST* genotype shows a significant effect of the genotype at *CAPN1* and the index based on *GYS1* found *PPP1R3A*. In other cases the clusters do not seem to belong to a common pathway. It seems likely that “mature size” can be affected by many pathways. Nevertheless, the pattern of effects across traits of SNP_lead_ 3 and 4 are so similar that there must be some biological connection. The SNP_lead_ 4 is close to *LCORL*/*NCAPG* (also found in human height studies) while SNP_lead_ 3 is *SLC16A11*/*ALOX12*/*TP53. SLC16A11* is a monocarboxylate transporter and it does not seem a good candidate for affecting mature size. *ALOX12* is arachidonate 12 lipoxygenase, which oxidizes arachidonic acid to a spectrum of bio-active lipid mediators.

Genes related to fat synthesis occurred in groups other than group 2, possibly because FA traits are over represented in our 56 traits. For instance, in group 5 are *PNPLA3*, *FADS2* and *EVOVL2*. When all 71 candidate genes in Table 6 are considered, 25 have a direct involvement in lipid metabolism. Even when the 8 genes in group 2 and these 3 genes named above are removed there are still 14 ‘lipid’ genes, many of which are involved in intra-cellular signalling using lipids, particularly the PI3K pathway. PI3K hydrolyses PIP3 to IP3 (Inositol 1,4,5-triphosphate) and DAG (1,2-Diacylglycerol), which are second messengers. The PI3K pathway connects extracellular signals, such as GPCR and typosine kinase receptors, to key molecules such as AKT and mTOR which integrate signals related to energy and nutrient status, and which in turn regulate many activities such as cell growth and cycling, apoptosis and glucose metabolism [32]. For instance, *PLCD4* (also found in human height studies) and *PLCB1* are phospholipases that cleave PIP2 (Phosphatidylinositol-4,5-Bisphosphate) into the second messengers IP3 and DAG (http://www.sabiosciences.com/iapp/ip3.html). ISYNA1 is IP3 synthase 1. *PROCA1* also has phospholipase activity. PI4K2B generates PIP4, a starting point for other PI messengers. The linear index derived from *PLCD4* was significant for a SNP close to *PRKAA2* that is a part of AMP kinase, which is important for energy homeostasis (Low ATP causes AMPK to decrease the activity of *ACACA* and *GYS1*, causing reduced FA synthesis and glycogen synthesis, respectively. *ACACA* is close to SNP_lead_ 6 and *GYS* to SNP_lead_ 17. ACACA is the rate limiting step for lipogenesis *de nova*.). AMPK connects to the PI3K pathway via Akt, which integrates many signals and in turn affects cell proliferation or apoptosis, and glucose metabolism. *GHR* and ErbB have numerous connections to the PI3K pathway. *LCAT* is a lecithin-cholesterol acyl transferase and *APOL6* is an apolipoprotein that transports cholesterol. *SGK2* is a kinase activated by signals that activate PI3K.

There are also a number of candidate genes associated with the cytoskeleton – *FRYL*, *MYO1H*, *MYO15. MYO15* was also found to affect growth traits in cattle. *FARP2* binds to both phospholipids and cytoskeleton and regulates integrin signalling and cell adhesion. *ARAP2* is a PIP3 dependent Arf GAP which regulates focal adhesion. The gene closest to SNP_lead_ 14 is laminin alpha 4 (*LAMA4*) which codes for a major protein in the basement membrane involved in cell adhesion and signalling and is related to the PI3K pathway. TBCK is thought to play a role in actin cytoskeleton organization, cell growth and proliferation via the mTOR pathway. AMPK, AKT and mTOR are critical controllers of energy use and protein synthesis.

Thus a hypothesis can be formed that the collection of 56 traits that we have analysed is controlled, in part, by cytokine signals (e.g. *GHR*) mediated by intra-cellular signalling pathways, especially PI3K, that control energy homeostasis, insulin sensitivity [33] and cell growth through effectors such as enzymes (e.g. *GYS*) and cell cytoskeleton changes. The gross effect of these pathways is that substrates get directed to different products (e.g. glycogen or FA) and eventually the balance of cell types (muscle fibre types or muscle vs fat) is affected.

Signalling systems within the cell are complex and inter-connected so it probably does not make sense to think of a linear pathway with all genes in the pathway having a similar phenotypic effect. Rather each gene has a unique position in a large network and therefore a unique pattern of pleiotropic effects. Nevertheless, the similarity of phenotypes of SNPs in group1 suggests that they must share some common parts of the network. The hypothesis put forward here is that this involves signalling, often via PI3K, to AMPK, AKT and mTOR.

### Application to sheep breeding

Sheep breeders are keen to improve the genetic merit of their sheep for carcass and meat quality traits. The pattern of effects of each QTL studied here indicates that some would be more useful for selection than others. Some QTL have an allele with desirable effects on more than one trait and appear to be good targets for selection. For instance, the QTL on OAR 2 (mapped near *PLCD4*) has an allele that increases tenderness, improves meat colour (i.e. increased redness of meat), increases myoglobin, glycogen, and unsaturated (omega-3 and -6) fatty acids and decreases saturated fatty acids, which is a highly valuable pattern. Selection for this allele would be beneficial in sheep intended for most markets.

## Conclusion

All traits appear to be highly polygenic with dozens to hundreds of SNPs (*P* < 10^−5^) across the genome independently associated with each trait. The FDR was lower in the multi-trait analysis than in single trait analyses, showing that it had increased power to detect significant associations with this group of traits implying that many SNPs are associated with more than one trait. The detailed phenotyping of 56 related traits helped to identify convincing candidate genes in cases where the phenotype was closely related to the primary action of the gene (e.g. FA synthesis genes). Cluster analysis arranged the significant SNPs into 5 groups so that SNPs within a group had a similar pattern of phenotypic effects. The genes near group 2 SNPs, which are associated with fatness and fat composition, are predominantly genes involved in FA and fat synthesis. By contrast, the genes near SNPs in group 1, which affect mature size, do not share a clear mechanism. However, these genes are also found in cattle and humans associated with size and fatness traits so it is unlikely that most of them are false associations. Rather it indicates our lack of understanding of the many processes that control mature size. Across the 5 groups there are many genes involved in lipid metabolism. These may act directly on measures of fatness but it seems likely that some of them are involved in signaling pathways within the cell. There was considerable overlap in the genes identified in our study and those reported to affect height and fatness in humans, and body composition in cattle. The incorporation of the identified causal mutations into genomic selection strategies could improve their accuracy and robustness, while allowing targeted selection to achieve more rapid genetic improvement.

## Methods

### Genotype data

This study utilised the Ovine Infinium® HD SNP BeadChip, comprising 603,350 (HD) SNPs (Illumina Inc., San Diego, CA, USA) and the Illumina 50k Ovine SNP chip (Illumina Inc., San Diego, CA, USA), comprising 54,241 (50k) SNPs. All SNP were mapped to the OAR 3.1 build of the ovine genome sequence assembled by the SNPchiMp v.3 [34]. The genotypes for each SNP of both 50k and HD SNP chips were encoded in the Illumina A/B format and then genotypes were reduced to 0, 1, and 2 copies of the B allele.

Stringent quality control procedures were applied to the SNP data. SNP were excluded if the call rate per SNP (this is the proportion of SNP genotypes that have a GC (Illumina GenCall) score above 0.6) was less than 95 % or minor allele frequency were less than 0.01 or an extreme departure from Hardy-Weinberg equilibrium (*P* < 10^−5^) occurred. Furthermore, if the average call rate per individual was less than 90 %, those animals were removed from the SNP data. Further details on quality control can be found in Daetwyler et al. [35]. The final set of our 50k SNPs consisted 38,942 SNPs and the sporadic missing genotypes of 10,613 animals were filled using the BEAGLE 4 program pedigree option [36].

After all the quality control tests were applied, 510,174 SNPs of the HD SNP chip remained on 1,735 animals and the sporadic missing genotypes were filled using FImpute [37]. Out of 10,613 animals with 50k and 1,735 HD genotypes, 1,682 animals were genotyped for both SNP arrays. The correlation between real 50k and HD genotypes for the 38,942 50 k-SNPs of these 1,682 animals was 0.9988.

The imputation of the 50k to HD was done using Fimpute [37]. All 1,735 HD genotypes were used as a reference set to impute from the 50k genotypes within each breed. Cross-validation within the 1,735 HD genotypes revealed an average accuracy of imputation (correlation of imputed empirical non-50k genotypes) of 0.9871. Most sires of phenotyped animals were genotyped at HD density. Thus, imputation accuracy in this study can be expected to be high as well. In total, 10,613 animals had real or imputed HD genotypes for 510,174 SNPs and a phenotypic records for at least one trait.

### Phenotype data and traits

The 10,613 animals (from 9 sheep breeds or populations including MER, PD, BL, SUF, WS, TEX, CORR, COOP, and MIX; Fig. 1) used in this study were sourced from the information nucleus flock of Cooperative Research Centre for Sheep Industry Innovation (**Sheep CRC**) and the SheepGENOMICS (**SG**) project [38, 39]. In total, 56 traits were measured (carcass weight, fatness, muscling, tenderness, meat colour, pH level, and fatty acid profile) and trait definitions, number of records for each trait, raw means and standard deviations based on the genotyped animals are given in Table 1. The pedigree file included 27,618 animals (including 1,236 sires and 9,638 dams) over 8 generations. A complete description of the design, methods and analyses of carcass and meat quality assessments is given by Mortimer et al. [4, 40]. Not all sheep were measured for all traits.

### Single-trait genome-wide association studies

#### Model used for GWAS

Mixed models fitting fixed and random effects simultaneously were used for estimating heritabilities and associations with SNP. The estimates of heritability were calculated based on pedigrees for all animals that have genotype and phenotype data. The same model was used for GWAS, except that each SNP (SNP_i_, i = 1, 2, 3, …, 510,174) was added to the model, one at a time, and tested for an association with the trait. The analysis was performed using the ASReml software [41] based on the following mixed model:

$$ \mathbf{y}={\mathbf{1}}_{\mathbf{n}}\mu +\mathbf{X}\mathrm{b}+{\mathbf{s}}_{\mathbf{i}}{\alpha}_{\mathrm{i}}+{\mathbf{Z}}_{\mathbf{1}}\mathbf{a}+{\mathbf{Z}}_{\mathbf{1}}\mathbf{Q}\mathbf{q}+{\mathbf{Z}}_{\mathbf{2}}\mathbf{d}+{\mathbf{Z}}_{\mathbf{3}}\mathbf{s}.\mathbf{f}+\mathbf{e} $$

where **y** is the vector of observed phenotypic values of the animals, **1**_**n**_ is an nx1 vector of 1’s (n = number of animals with phenotypes), μ is the overall mean, **X**, **Z**_**1**_, **Z**_**2**_, and **Z**_**3**_ are all design matrices relating observations to the corresponding fixed and random effects, **b** is a vector of fixed effects (described below), **a** is a vector of polygenic additive genetic effects sampled from the distribution N ~ (0, **A**σ_a_^2^), where σ_a_^2^ is additive genetic variance and **A** is the additive relationship matrix constructed from the pedigree of the animals and their ancestors, **q**, **d**, **s.f**, and **e** are the vectors of random effects of breed (including Merino strains), dam (permanent environment), sire by flock interaction, and residual error, respectively. **Q** is a matrix with breed and strain proportions calculated from pedigree (q ~ *N* (0, **Q**σ_q_^2^) [42]; **s**_**i**_ is a vector of the genotype of each animal at the *i*th SNP, **s**_**i**_ is fitted as covariates. The maternal group and/or sire by flock interaction were significant (*P* < 0.05) to be included in the model for carcass weights, fatness, and muscling traits, whereas it was not significant for tenderness, IMF, meat colour, pH level, and fatty acid traits.

All models included dataset (or project) origin (Sheep CRC and SG), management group, flock, date of observation, birth year, sex, birth type, and rear type as fixed effects. All fixed effects were fitted as nested within a dataset. Flock, date of observation and birth year were combined in one contemporary group. Also, birth type and rear type were grouped together. Also, the laboratory effect was fitted as a fixed effect, and it was significant (*P* < 0.05) only for shear force and FA traits. Carcass traits, excluding HCWT, DMLION and IMF, were corrected for HCWT, and saturated FA traits were corrected for IMF. Age of dam and its square and age at observation were fitted as covariates. Age of dam ranged between 1 and 9 years. The age at observation varied from 134 to 705 days.

### Significance of SNP effect

SNP were tested for a significant association with particular traits at different probability thresholds (Table 2). Following Bolormaa et al. [43], the false discovery rate (FDR) was estimated as $\frac{P\left(1-\frac{A}{T}\right)}{\left(\frac{A}{T}\right)\left(1-P\right)}$ where *P* is the *P*-value tested (e.g. 0.00001), *A* is the number of SNP that were significant at the *P* -value tested and *T* is the total number of SNP tested.

### Validation of SNP effects

In order to validate statistically significant SNP effects in an independent population, the animals with phenotype and genotype data for each trait were split into five sets by allocating all of the offspring of randomly selected sires to one of the five datasets. Then one of the 5 divisions was randomly used as a validation population and the other 4 divisions as the reference population. Only one 4:1 division of the data was used per trait. In this way no animal used for validation had paternal half sibs in the reference population. The GWAS for 4 traits, which are amongst the traits with the highest number of significant associations, were performed in the reference population (Table 3). For SNPs with a significant effect in reference population, the analysis was repeated in the validation population. We counted the number of times that the estimated SNP effect was in the same direction in the validation population.

### Multi-trait analysis to detect pleiotropy

#### Multi-trait significance test

Multi-trait, meta-analysis, following the procedure in Bolormaa et al. [17] were performed based on SNP effects estimated from 56 individual single-trait GWAS. The multi-trait χ^2^ statistic was calculated as: multi-trait χ^2^=, where ***t***_***i***_ is a 56 × 1 vector of the signed t-values of SNP_**i**_ effects for the 56 traits, ***t***_***i***_’ is a transpose of vector ***t***_***i***_ (1 × 56), and *V*^−1^ is an inverse of the 56 × 56 correlation matrix where the correlation is calculated over the 510,174 estimated SNP effects (signed t-values) of the two traits. The power of QTL detection was investigated by comparing FDR [43] calculated in the multi-trait test with FDR [43] calculated in the single-trait GWAS (Table 3).

#### Use of linear indices in multi-trait validation

A linear index of 56 traits that had the maximum correlation with genotypes for significant SNP was used for multi-trait validation. The linear index on individual animals could only be calculated for animals with all traits measured. Not all animals were measured for all traits, so missing values were filled in by a prediction using a multiple regression approach as described by Bolormaa et al. [44]. Using this approach, the actual effects (not signed t values) of 510,174 SNPs for 56 traits that were estimated based on all animals were used in order to have the same units as the phenotype values. Before the missing phenotypes were predicted, the raw phenotypes for each trait were corrected for fixed effects using the following model: corrected phenotype = phenotype − fixed effects. Please note that missing phenotypes were not predicted for single trait GWAS and the multi-trait significance test above.

As a validation, after filling missing values, all data was split into discovery and validation populations using same approach described in the single-trait GWAS section. Then the individual trait GWAS and the multi-trait significance test on signed t-values described in the previous sections were performed using only the discovery population (only one 4:1 division of the total data was used per trait). Only the most significant SNPs (*P* < 10^−5^) within a 1 Mb window (to avoid testing a large number of closely linked SNPs) from the multi-trait analysis in the discovery set were validated in an independent set of animals. After this, a linear index (*y*_*I*_) of 56 traits that had maximum correlation in the discovery population with each selected (significant) SNP was calculated using the following formula [43]: *y*_*I*_ = *b* ' *C*^− 1^*y*, where *b*’ is the transpose of a vector of the estimated effects of the SNP (not t values) on the 56 traits (1 × 56) that was estimated from only the discovery population, *C*^−*1*^ is an inverse of the 56 × 56 (co) variance matrix among the 56 traits calculated from the estimated SNP effects of 510,174 SNPs only in the reference population, and y is a 56 × 1 vector of the phenotype values for 56 traits for each animal in the validation sample. The association between each linear index (*y*_*I*_) and each SNP (only significant SNPs in every 1 Mb window from GWAS in discovery population) was then tested in the validation population. The *y*_*I*_ was treated as a new trait (dependent variable). The association was assessed by a regression analysis (GWAS) using the following model: *y*_*I*_ ~ mean + SNP_i_ + animal + error, where animal and error were fitted as random effects and SNP_i_ were fitted as a covariate one at a time (other fixed effects were removed from the trait measurements before forming the linear index). In order to see whether the SNPs validated in the validation population have the same direction of effects (positive or negative) as SNPs in the discovery population, we also calculated linear index and performed linear index GWAS by using the phenotypes of the discovery population instead of the phenotypes of the validation population. Then the directions of SNP effects for the linear index in both reference and validation populations were checked and the proportion of SNPs whose effects were in the same direction in the reference population was calculated.

### Conditional analyses accounting for 23 lead SNPs

The 23 SNP_lead_ were selected as follows: On each chromosome the most significant SNP (*P* < 10^−5^), based on the multi-trait analysis, was selected. Up to three SNPs on the same chromosome were selected only if they were at least 4 Mb apart and represented two or three different QTL (showing clearly differentiated peaks on Manhattan plot from the multi-trait analysis).

The regression analyses (GWAS) were repeated and, additionally, the 23 SNP_lead_ were fitted simultaneously in the model. The statistical model used was the same as in single-trait GWAS with an addition of the 23 SNP_lead_, which were fitted simultaneously as covariate effects. Then a multi-trait chi-squared statistic was calculated for each SNP to test the effects of the SNP across traits after fitting the 23 SNP_lead_.

### Cluster analysis to find QTL with a similar pattern of effects across traits

The SNP effects estimated from single-trait GWAS based on all animals were used to investigate the relationships between SNPs. For each pair of SNPs among the 23 SNP_lead_, the correlation of their effects across the 56 traits was calculated. Highly positive or negative correlations indicate 2 SNPs with the same pattern of effects across traits. Then this correlation matrix was used to do the hierarchical clustering of the 23 SNP_lead_ leading to 5 groups or clusters as shown in the dendrogram drawn using the heatmap function of the R program [45].

### Searching for more QTL in the same pathway using linear indices of SNP_lead_

For each of the 23 SNP_lead_, we searched for additional SNPs in the 5 groups defined by the cluster analysis. To do this we used the linear index that showed the highest association with the corresponding SNP_lead_ genotype, as previously defined for validation of the multi-trait analysis. The linear index of traits that had a maximum correlation with the genotypes for each of 23 SNP_lead_ was calculated based on all data. A new GWAS was performed for each of 23 linear indexes (*y*_*I*_) treating it as a new trait (dependent variable). To avoid identifying QTL already represented by the 23 lead SNPs, the 23 SNP_lead_ were also fitted simultaneously in the model. In this way, we could discover new QTL which are associated with one of the linear indices corresponding to the 23 SNP_lead_. The following model was used: ~ mean + fixed effects + SNP_i_ + leadSNP_1_ + leadSNP_2_ + … + leadSNP_23_ + animal + error, where animal and error were fitted as random effects and the *i*th SNP (SNP_i_, i = 1, 2, 3, …, 510174) and 23 SNP_lead_ were fitted simultaneously as covariate effects.

The SNPs that have significant associations (*P* < 5 × 10^−7^) with at least one of the indexes based on SNP_lead_ were selected for assigning into 5 groups. These additional significant SNPs were assigned to the same group as the SNP_lead_ with whose linear index they had the most significant association.

### Identifying plausible candidates

The genes that occur within 30 kb of the SNPs in this expanded list of significant SNPs were identified using UCSC Genome Bioinformatics (http://genome.ucsc.edu/) and Ensembl (www.ensembl.org/biomart/). If there were more than one gene within 2 Mb, then only one gene was retained within the 2 Mb regions by selecting the nearest to the SNP or the particular gene has concomitant relationship with SNP effects associated for traits studied. GO and KEGG analysis in STRING (Search Tool for the Retrieval of Interacting Genes/Proteins) network program [22] was used to identify functional similarity between the genes near group 2 SNPs.

### Do these candidate genes affect similar phenotypes in other species?

30 kb upstream and downstream of 184 genes associated with fatness in humans were examined in the multi-trait sheep GWAS and found to contain 137 significant SNPs (*P* < 10^−3^) in 55 genes associated with fatness in humans. To test if this degree of overlap could be due to chance we randomly selected 184 genes and performed the same analysis. This was done 1,000 times to establish the distribution of the number of significant SNPs and genes under the null hypothesis that the genes that are significantly associated with fatness in humans are no more likely to contain significant SNPs in sheep than expected by chance. To test the results for genes associated with human height, a similar test was performed using 494 randomly selected genes.

### Availability of supporting data

The data sets supporting the results of this article are available in the additional files (Additional files 4, 5, 6, and 7).

### Ethics approval and consent to participate

Animal Care and Use Committee approval was not obtained for this study because no new animals were handled in this experiment. The experiment was performed on trait records and DNA samples that had been collected previously.

## Additional files

Additional file 1: Figure S1. Multiple plots of the –log_10_ (*P*-values) of SNP effects from the multi-trait test results for lead SNP OAR6_37.5 Mb (a) and OAR22_20.3 Mb (b): The lead SNP is shown by a purple diamond in each plot (labelled with chromosome and position, Mb) and the LD between this variant and all others is colour coded. (TIFF 322 kb) Additional file 2: Figure S2. Network graphic showing the interactions between Group 2 gene encoding proteins: Stronger associations are represented by ticker lines [22]. (TIFF 281 kb) Additional file 3: Table S1. A list of the overlapped candidate genes in QTL regions from our sheep study with genes associated with body mass index and height in human. (XLSX 15 kb) Additional file 4: The signed t-values of SNP effects (1 to 150,000 in “chromosome: position” order) for the 56 traits used in multi-trait, meta-analysis. (GZ 30545 kb) Additional file 5: The signed t-values of SNP effects (150,001 to 300,000 in “chromosome: position” order) for the 56 traits used in multi-trait, meta-analysis. (GZ 30615 kb) Additional file 6: The signed t-values of SNP effects (300,001 to 450,000 in “chromosome: position” order) for the 56 traits used in multi-trait, meta-analysis. (GZ 30638 kb) Additional file 7: The signed t-values of SNP effects (450,001 to 510,174 in “chromosome: position” order) for the 56 traits used in multi-trait, meta-analysis. (GZ 12388 kb)
