# Supplementary figures and images for: Detailed phenotyping identifies genes with pleiotropic effects on body composition
Source: BMC Genomics. 2016 Mar 12;17:224. doi: 10.1186/s12864-016-2538-0 (PMC4788919; doi:10.1186/s12864-016-2538-0)

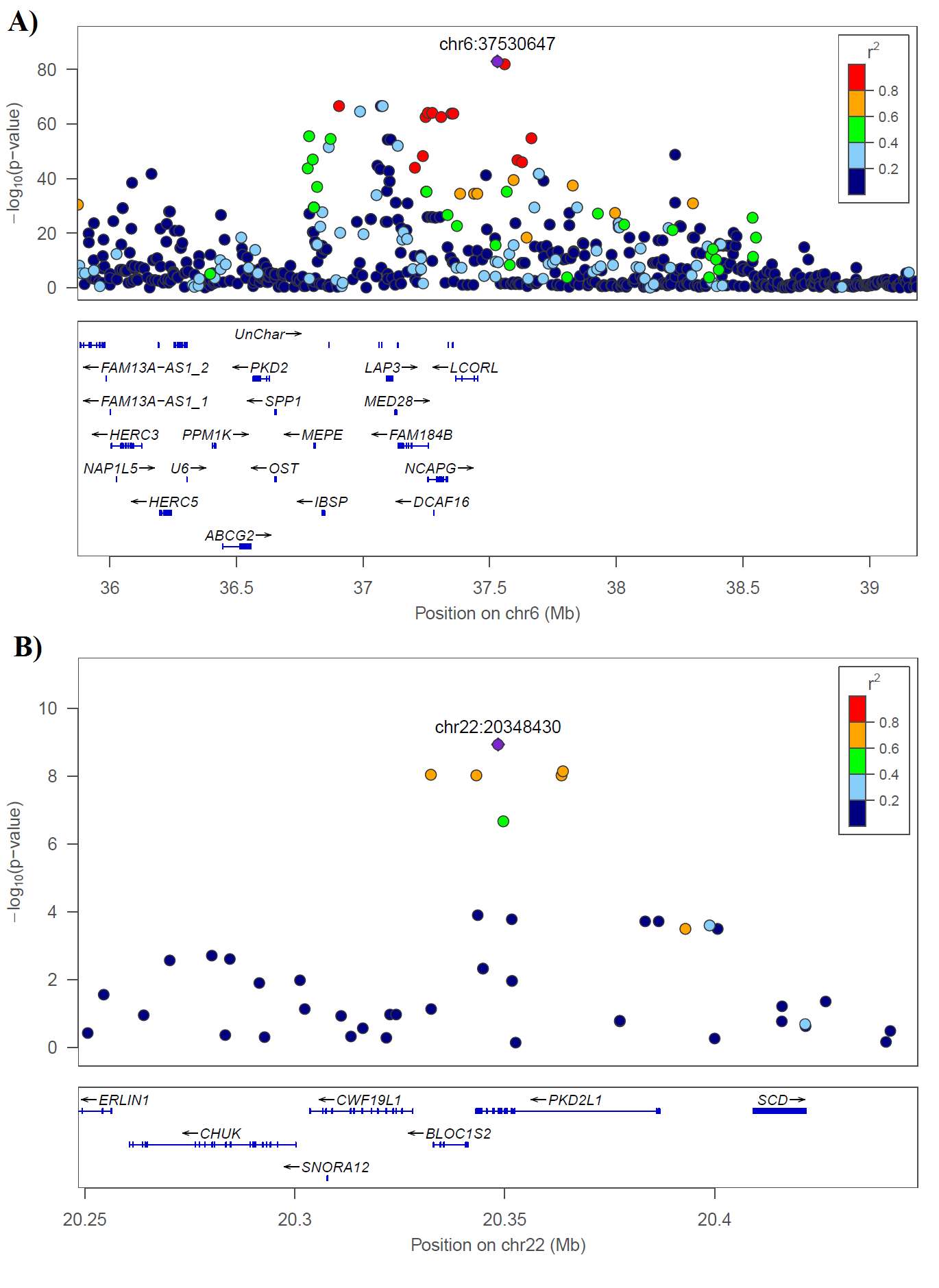

Supplement: Additional file 1: Figure S1. — Multiple plots of the –log10 (P-values) of SNP effects from the multi-trait test results for lead SNP OAR6_37.5 Mb (a) and OAR22_20.3 Mb (b): The lead SNP is shown by a purple diamond in each plot (labelled with chromosome and position, Mb) and the LD between this variant and all others is colour coded. (TIFF 322 kb) [file 12864_2016_2538_MOESM1_ESM.tiff]

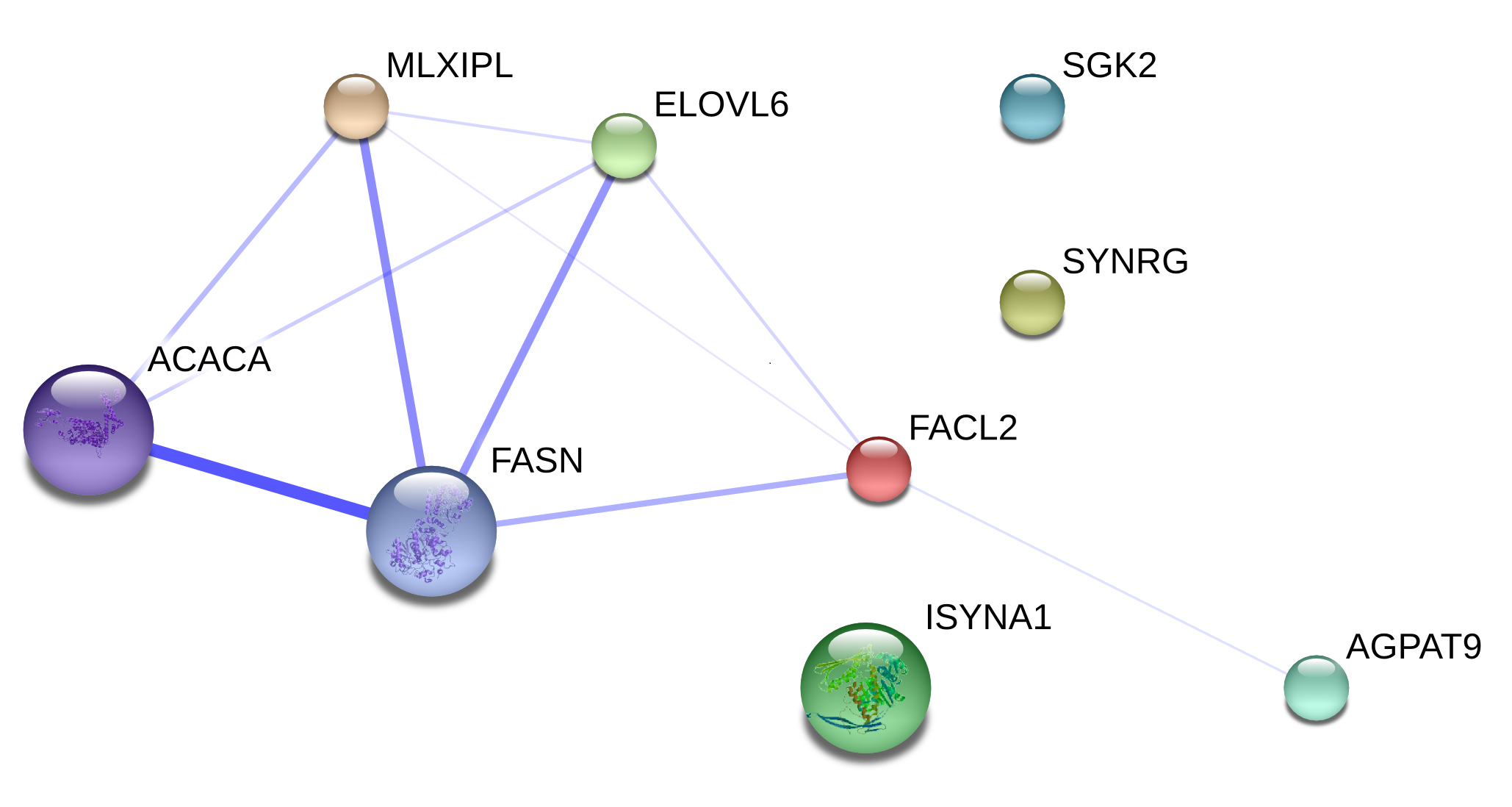

Supplement: Additional file 2: Figure S2. — Network graphic showing the interactions between Group 2 gene encoding proteins: Stronger associations are represented by ticker lines [22]. (TIFF 281 kb) [file 12864_2016_2538_MOESM2_ESM.tiff]
